# Postoperative anaemia increases unplanned readmission: an international prospective cohort study of patients undergoing major abdominal surgery

**DOI:** 10.1093/bjs/znae158

**Published:** 2024-07-11

**Authors:** Kenneth J Macpherson, POSTVenTT International Collaborative, Kenneth J Macpherson, Ane Abad Motos, Vinicio Mosca, Ruth Blanco Colino, Muhammed Elhadi, Eman Ali, Cameron Wells, Chris Varghese, William Xu, Jurstine Daruwalla, Gianluca Pellino, Jose Antonio Garcia Erce, Nagendra N Dudi-Venkata, Toby Richards, David I Watson, Pete Pockney, Deborah M Wright, Doug Robb, Guiliana D’Aulerio, Jani Lee Moss, Uyen G Vo, Jai Darvall, Matthew A Warner, Warren Seow, Laure Taher Mansour, Jayne Lim, Su Kah Goh, David S Liu, Muhammad Ibrahim, Amanda C Dawson, Elizabeth W Y Lun, Adam J Frankel, Siang Wei Gan, Jonathan Foo, Francesco Pata, Liam Ferguson, Daniel Chubb, Christophe Berney, Sonalmeet Nagra, Chonhann Liew, Hasanga Jayasekera, Merwe Hartslief, Adeep Majid, Henry Y Cheung, Sayed Hassen, John VanBockxmeer, Tobias J Evans, Francesco Amico, Girish Pande, Justin S Gundara, Elizabeth Murphy, Bernd Froessler, Sarah Martin, Daniel Croagh, William Teoh, Matthias Wichmann, Jonathan Fong, Krinalkumar Mori, Quentin Ralph, Aliakbar Estakhri, Sarah R Bowman, Harsh Kanhere, Benjamin Loveday, Stephanie Chetrit, Dale Currigan, Mary Theophilus, Michael J Johnston, Markus Trochsler, Justin Yeung, Guy Maddern, Richard G McGee, Mohamed Asal, Mostafa Abbas, Giuseppe Spampinato, Donato F Altomare, Piergiorgio Danelli, Andrea Mingoli, Simone Giacopuzzi, Luca A Fumagalli, Stefano Olmi, Marzia Franceschilli, Adolfo Pisanu, Ghaith Bani Abdel Rahman, Milad Gahwagi, Ian Bissett, Jeremy Rossaak, Saxon Connor, Alexander W Brown, Andrew MacCormick, Sze-Lin Peng, Peter Stiven, Bevan Jenkins, James Tietjens, Christopher Harmston, Christopher M Gray, James G Wilkins, Jasen Ly, Andrew Herd, Sarah Rennie, Marianne Lill, Raed Amro, Jose Ramon Rodriguez Fraile, Maria Moral Gonzalez, Santiago Valderrabano-Gonzalez, Montasir A Abdalbain, Ahmad Ghazal, Nashwan Tashan, Mohammed Al Shehari, Jed Hughes, Joseph C Kong, Melanie E Cusso, Daniel R Cox, Sarit S Badiani, Sara Mohammed Jinnaah, Amy Crowe, Enoch Wong, Melissa Y Wee, Matthew J Marino, Isabella Ludbrook, Anne-Marie Aubin, Timothy J Ganguly, Tara Kannan, Fiona Pavan, Geraldine J Ooi, Vignesh Narasimhan, Paul T Heitmann, Tiffany J Cherry, Sharon Lee, Teng-Wei Khoo, William Lindores, Christine Li, Xiao-Ming S Woon-Shoo-Tong, Amelia L Davis, WeiShearn Poh, Thomas Tiang, Victor W Chai, Gayatri Asokan, Eunice Tse, Brianne Lauritz, Alicia Lim, Mahmoud Shaban Abdeljalil, Moataz Ewedah, Ahmed Abbas, Marian Abou El Garad, Hossam Elfeki, Mohamed Elbahnasawy, Stefano Garritano, Arcangelo Picciariello, Francesco Colombo, Pierfrancesco Lapolla, Paola De Nardi, Mauro Zago, Matteo Uccelli, Giuseppe Sica, Mauro Podda, Almu’atasim Khamees, Wafa Aldressi, Eman Abdulwahed, Jelle M Vander Have, Imogen I Watt, Brodie M Elliott, Chelsea A Allen-Brough, Carys Finlayson, Olga Korduke, Mathew Morreau, George N Weeratunga, Matthew J McGuinness, Jethro Palmer, David C Kieser, Xavier Field, Dhenisha Dahya, William Xu, Louis-Antoine Bonnet, John George Grace Massoud, Sarah Amro, Fátima Cañeque Yanini, Beatriz Amoros, Aranzazu Calero Lillo, Misericordia Basora, Rosalia Navarro Perez, Rita Pilar Rodriguez Jimenez, Carmen Deiros Garcia, Aitor, Cesar Aldecoa, Montse Mallol Oliva, Raquel Garcia Alvarez, Virginia Jiménez Carneros, Raquel Fernandez Garcia, Hanna Perez-Chrzanowska, Maria Vila Montanes, Maria Luisa Pinana Campon, Hytham K S Hamid, Ruqaya Masri, Abdulrahman Almjersah, Ali Alloush, Fatima Al Eryani, Jonathon Holt, Stephanie Potenza, Avelyn Wong, Sagarika Handa, Virginia Su, Aniqa Hussain, Angus Tolhurst-McKiernan, Amelia J Cooper, Mark T Butorac, Hugh Elbourne, Emily Dutta-Powell, Neave Kapoor, Caitlin Reid, Jason Wang, Stella Le, Jordi Elliott, Bill Wang, Jiting Li, Zhuoning Song, Aya Basam, Branavan Thevashangar, Shani Nguyen, Arsheeya Rattan, Jeffrey Shao, Cynthia Wells, Emma Aldous, Siobhan Brodrick, Brooke Callahan, Faizah Alam, Laura Casey, Wilson Yang, Swetha Prabhakaran, Haini Jiang, Alex Lin, Angela Chau, Pranjal Chaurasia, Morgan Jones, Yit J Leang, Wendy Brown, Jonathan Serpell, Peter Carne, Matthew Stokes, Jern Cabral, Miriam W Bennamoun, Suneha Lal, Kai Dong Choong, Yanran Yu, Chris Zhao, Vijayaragavan Muralidharan, Sean Stevens, Liam Fernando-Canavan, QiRui Soh, Nakjun Sung, Grace Gold, Alex Grogan, James G Lin, Ahmad Aly, Georgina Riddiough, Hamza Ashraf, Michaela Wojno, Adrian Yeoh, Gabriel Wall, Jack Liu, Sonia Gill, Yida Zhou, Robert Torode, Michael Issa, Jasun Li, Ruben Schuringa, Marcos V Perini, David Proud, Kevin Jia, Suhasini Shanmugarajan, Gladys Tam, Ashley Wang, Shipra Sankpal, Matthew Heard, Yung-Hsin Hsueh, Scott Anderson, Vishwakar Panuganti, Zoe Zhang, Gabriel Lirios, Joshua Birnie, Benjamin Scott, Lei Ying Barwon, Laura LeHua He, Samantha Mitchell, Chloe Wu, Brett Larner, Jason Fu, Amy McCormick, Daryn Goodwin, Alistair Lau, William Stockley, Ripudaman Jalota, Hannah Gora, Parvathy Ananthan, Thomas Estephan, Aakash Nanda, Sarika Suresh, Thihoai Trang Nguyen, Sarah McLain, Sharanya D Mohan, Nicole Batten, Gajanth Sivapalan, Grace S Ting, David Yoon, Joohyen Ahn, Lisa Shi, Salena Ward, Felicity Huang, Boya MacKeddie, Adrian Fox, JiYue Wang, Amie Hilder, Ashan Kathriachchige, Chen Lew, King Tung Cheung, Sarah Goh, Balakavitha Balaravi, Simran Vinod Benyani, Chris Ip, Shomik Sengupta, Christopher Steen, Janindu Goonawardena, Shantanu Joglekar, Erica Wang, Olivia Lin, Michelle Yao, Vinna An, Connor Burley, Raaj Chandra, Anshini Jain, Amelia Davies, Matthew Mann, Anand Trivedi, Matthew J Pugliese, Ron Ramos, Davina Daudu, Tasvinder Hans, Audrey Kim, Aiden Stuart, Harvinder Hans, Natalie Layden, Pravin Viswambaram, Ashley Lee, Mohammed Al-Zubaidi, Dickon Hayne, Hayley J McMillan, Jason D’Silva, Ben P Finlay, Rebecca Fong, Penelope Peres, Gavin Nair, Suchitra KrishnanPillai, Yong Zhi Beh, Steven L Due, Benjamin Howes, Dominic Parker, Christina Mcveay, Matthew Marshall-Webb, Izhar-Ul Haque, AbdulAhad Rana, Sneha E Ninan, Gemma J Qian, Joseph W Fletcher, Brendan Watkins, Luke C Green, Yara A Elgindy, Elodie V Honore, Charles J Roth, Jonathan Tandjung, Adam R Perkovic, Luca Borruso, Victor Yu, Thomas-Hoang Le, Samuel M Mathias, Daniel Jeong, Charlotte E Cornwell, Emily A Taylor, Kie Ren Oon, Mikayla Everuss, Jesse Hafner, Caleb Kim, Catherine T Cartwright, Aram Cox, Ricci Amolis, Hui Kang Tan, Brittany Harrop, Theepiga Ramachandran, Elli Farrell, Saxon Farquhar, Felicity Goodes, Sophie Zhong, Sophie Kelly, Howard Li, Jun Jie Chau, Joshua Richards, Christopher Cheong, Tedman Chau, James Pankhurst, Darren J Rama, Alicia Liong, Sebastian Primrose, Peita M Webb, Edward Young, Harleen Kaur, Xuanyu Zhou, Mohamed Afzal, Jess Barnes, Allan Butler, Priya Nandoskar, Matt Besley, Mozafer Rajabali, Nikhil Kundu, Alexander Buckby, Linda Sunny, Olivia Anderson, Hemachalam Subramaniam, Sandeep Dayaratne, Anjelee Segaran, Daniel Chilton, Chris J Kim, Dion Koh, Tom A Eldredge, Syeda T Saief, Kevin K Yoong, Ashray Rajagopalan, Kathleen Wisbey, Chelsea Lin, Jolene Ong, Sandra Li, Jonathan Tiong, Naman Kohli, Joseph Jaya, Jiakai Yao, Evie Yeap, Katherine Grant, Genevieve Heath-Kalgutkar, Ryan R Gosavi, David Zula, Ning Yih Kam, Travis Ackermann, Darryl Lee, Sean Lim, Alice Lee, Phi Nguyen, Lily Bae, Roshini Nadaraja, David Duong, Emma Payne, Natalie Nguyen, Gabrielle Cutler, Fellicia Stanzah, Matthew Watson, Luke Green, Aveechal Prasad, Laxmi Sistla, Emily Overton, Monika A Wojt, Amos Moody, William Zore, Rebecca Blundell, Tarik Sammour, Syeda Khadijah Ahmed, Yickho Lam, EuLing Neo, Anthony Farfus, Leigh Warren, Zachary Bunjo, Jaspreet K Sandhu, Qiwen Wang, Syeda Khadijah Ahmed, Nimantha Abey, Victoria Lu, Amanda Hii, Shalvin Prasad, Christopher Dobbins, Mohammad Mangi, Joseph Dawson, Danielle Boniface, Mathew Amprayil, Patwinder Gill, Jonathan McCafferty, S F Michelle Ku, Zijiang Yang, Ming Z How, Frank Du, Reece Ansaar, Chelsea Xu, Nyan S Lwin, Supisara Suk-Udom, Nathan Scott, Kyle Raubenheimer, Nivedan Jeyamanoharan, Priyanka Chakraborty, Bryce Stewart, Dinethma Tharuni Ekanayake, Diana Xiao, Aiswarya Ajith, Mark Ibraheem, Joseph Hanna, James Dodd, Sophie Zhao, Aine O’Dwyer, Sinali Seneviratne, Lauren Masi, Hilary Leeson, Xiao-Ming S Woon-Shoo-Tong, Ho-Cing Yau, Daniel Lee, Siavash Mortazavi, Stephanie Bartley, Zheng DaChok, Nur Sabrina Binti Babe Azaman, Leesha Bryan, Bethany Furfaro, Peng Sheng Tan, Talia Shepherd, Mary Teoh, Kelly Shepherd, Danielle Malatzky, Nicole Hew, Pia J Schelfhout, Rama Chidambaram, Ashwita S Vanga, Madiha K Bajwa, Aidan D YueOye, Darren Karadimos, Nyssa Jualim, Vivien Hsu, Sharie A Apikotoa, Cathy Maher, Emalee Burrows, Natalie Yu, Michael W Hii, Lillian Taylor, Lynn Chong, Louis J Sisk, Cathy Hua, Elaine Su, Aidan Jackson, Elizabeth Hu, Tori Wong, Andrew Xu, Toni Zhang, Luke Bradshaw, Kathleen Nagle, Antonio Barbaro, Lata Limbu, Mai Nguyen, Kalyanasundaram, Colin Choi, Ha M Nguyen, Shyamolie Mathur, Nicholas Quirk, Carol Z Wu, Reshi Suthakaran, Chui Foong Ong, Ali Baker, Rosalina Lin, Jade T Bahnisch, Haili Luo, Mohamed Qassem, Omar Saeed Anas, Omar Abdelnasser Mohamed, Basil Alshami, Mariam Kefah Fathi, Muhammed Ahmed Muhammed Abdelrazeq, Hajar Fawzy Abdel Rady, Mahmoud El Garhy, Laila Raafat Mabrouk, Balqees Alhariri, Ahmed K Awad, Merihan A Elbadawy, Mahmoud Elsayed Abdelazeem Bakheet, Nadien Mohamed Abd El Hamid, Noha Eslam Attia Sakr, Yara Mohamed Yasser, Mostafa Osama Farag, Selwan Mahmoud Abdelrahman, Israa Mohamed Iraqui, Dalia Shaban Abdelgalil, Sherein Diab, Peter Nader Sobhi Rafla, Steven Danial Azmy Habib, Aya Abdulilah Abdulmonem, Bassant Hassan Shawki, Salma Adel Mohamed, Sally Ahmed Abd El Razik, Ahmed Hassan A Rady, Ayatallah Ali Hussein Ramadan, Ayman K Awad, Pavly Wagih Beshay, F R S Gad, Salma Tarek Helmy, Michael Amin, Mahmoud Mohamed Mohamed Shalaby, Abanoub Ashraf Moufid Riyad, Ahmed Nafea, Marina Hany, Ahmed Sakr, Mohamed Al Sayed, Mosaab Tayiawi, Mohammed Eleleigy, Osama Elzankaly, Mohamed Mourad, Shaher Shokralla, Yomna Dean, Yousef Tanas, Samar Abdelnaby, Abdulrhman Abdulhameed, Afnan Morad, Esraa Emad Abdelazeem, Manal Abdelghany, Yosra Rateb, Eithar Alqady, Shereen Elnokrashy, Mona Helbawi, Asmaa Shaltout, Ahmed Hussein, Amira Salem, Ramy Hassan, Magdy Mahdy, Marina Adly, Sarah Abdelmoneim, Ahmed Taha, Ashraf Elnaggar, Mohamed Elkady, Omar Soliman, Ahmed Aboelmagd, Mahmoud Shehata, Shymaa Abodahab, Rehab Mohamed, Ahmed Youssef, Radwan Torky, Mohamed Abulfetouh, Gihan Galal, Sara Kalaifa, Mahmoud Khalil, Islam Ibrahim, Ahmed Kamel Morsi, Ahmed Wahba, Islam Abo Deef, Ahmed Abd Elzaher, Alaa Sultan, Abdallah Rashad Temerik, Ahmed Abdelmoneim, Shereen Saad, Shaymaa Ibrahim, Ahmed Elkoussy, Yasmine Adel Mohammed, Roaa Saleh, Randa El Zohne, Mohamed Shaker, Fatma Gharib, Esraa Kotb, Eman Hassan, Islam Aziz, Mohamed A Abdelmaksoud, Ahmed Ramadan, Reem Elsaadany, Mohamed Taman, Karim Elkenawi, Esraa Daif, Hossam Elsonny, Rahma Elboraei, Mahmoud Laymon, Osama Ezzat, Ahmed Elbatta, Mohamed Hassan, Amr A Elsawy, Ahmed Elsheshtawy, Islam Mansour, Amgad Fouad, Amr Sanad, Amr Kassem, Ahmed Beah, Ahmed Shehta, Ahmed E Nabih, Rana Nagi, Mostafa Shalaby, Ahmed Sakr, Yousef Hesham, Sameh Emile, Aly Sanad, Ahmed Elsayed, Mirna Sadek, Omar Taha, Shady Elsalhawy, Mustafa Zaghloul, Olli Helminen, Heikki Huhta, Matti Aleksi Mosorin, Henri Juhani Sova, Ville Virta, Norma Depalma, Gabriella Lionetto, Stefano D’Ugo, Tiziana Marchese, Farshad Manoochehri, Federico Perrone, Rigers Dibra, Giuseppe Trigiante, Gennaro Martines, Luciano Rella, Marco Vulpi, Pasquale Ditonno, Giuseppe, Francesco Cammarata, Luca Ferrario, Andrea Bondurri, Alessandro Michele Bonomi, Anna Maffioli, Isabella Pezzoli, Albert Troci, Franco Palmisano, Andrea Gregori, Giacomo Piero Incarbone, Giancarlo D’Ambrosio, Andrea Picchetto, Elena De Stefani, Gioia Brachini, Flavia Ciccarone, Ilaria Clementi, Giuseppe Marenga, Piero Bartolucci, Gennaro Mazzarella, Bruno Cirillo, Immacolata Iannone, Cristina De Padua, Enrico Fiori, Paolo Sapeinza, Simona Meneghini, Martina Zambon, Giulia Duranti, Costantino Leonardo, Giorgio Franco, Pierfranco Maria Cicerchia, Luigi Simonelli, Giovanni Battista Fonsi, Sara Giovampietro, Edoardo De Meis, Monica Mansi, Giovanni Casella, Lidia Castagneto Gissey, Anna Falasca, Catia Gugliuccello, Barbara Binda, Maura Rocchetti, Sara Santillo, Federica Scarno, Andrea Vignali, Simonetta Massaron, Marco Ee Chiarelli, Fulvio Tagliabue, Alessia Malagnino, Giovanni Pesenti, Carmine Sciorio, Alessandro Maletta, Michele Morelli, Antonio Pellegrino, Claudio Reato, Giani Sofia, Giusi Giulia La Manna, Andrea Martina Guida, Bruno Sensi, Vittoria Bellato, Giuseppe Tisone, Roberta Angelico, Tommaso Maria Manzia, Leandro Siragusa, Giuseppe Farullo, Luca Orecchia, Lorenzo Alteri, Virgilio Michael Ambrosi Grappelli, Tiziana Pilia, Valentina Murzi, Gianmarco D ancona, Stefano Angioni, Monica Ortenzi, Maurizio Nicola D’Alterio, Alessandro Cois, Marcello Pisano, Luai Abu Ismail, Younis Maher, Abdallah Yasin, Aseel Almohtasib, Farah Al Kasaji, Saja Aloran, Lana Sbitan, Heba Abu Khalaf, Subhi Al Issawi, Radi Tofaha Alhusseini, Sajeda Awadi, Issa A Abuzeid, Sarah Aldressi, Mohammed Alabeedi, Mohammed Moftah, Eman Salem, Mohammed N Albaraesi, Haitham Elmehdawi, Ahmed Ahmayda, Ala Burgeia, Tarik Eldarat, Maruwan Safi Abdullah, Abraheem Aljibali, Rema Benhariz, Almotasem Bellah Elsharif, Yasmin Abu Ihlayqah, Intesar Elmejrisi, Fatimah Abouthiheer, Rabha Ibrahim, Ayoub N Abdalmaged Akwaisah, Suha Younis Hasan, Ehsan Al Mahdi, Ehtuish Farag Ehtuish, Mohamed Alsori, Sultan Ahmeed, Sanad Kanna, Entisar Alshareea, Enas Soula, Sarah Aljamal, Ehda Kerwash, Doaa Gidiem, Ekram Abujrad, Hayat Ben Hasan, Aamal Askar, Esmail Hamuda, Khawla Derwish, Reem Ghmagh, Abdulqudus Deeknah, Fras Elhajdawe, Marwa Sinan, Jim Hsu-Shun Wang, Binura Lekamalage, Arpita Bansal, Luke DeRuiter, Kevin Yi, Faseeh Zaidi, Cindy Xu, Cain Anderson, Gabrielle Murti, Natasha P Mitchell, Celine Peng, Olivia Mackay, Daniel A Carson, Sameer Bhat, Jenny Choi, Justin Roake, Oliver Lyons, Eric T Lim, MeiSze Lee, Jared McLauchlan, Claire Whitehead, Sandra J Campbell, Fiona Carey, Grace Gatenby, Angharad Jones, Tamara Glyn, Heidi Blackburne, Emilie Roberts, Lauren Siggins, Renato Pitesa, Braedon Epps, Emma Espiner, Jack Hanlon, Ben Waller, Abhinav Bhasin, Sharmaine Sreedhar, Lauren Hubbard, Emily Evison, Molly Reynolds, Harriet Marshall, Sam Guest, Hinerangi Temara, Alexa Kuffel, Wing Yung Agnes Chu, Rebecca Teague, Rowan Auchterlonie, Vilingatoni Hasiata, Benjamin Keren, Joshua Tiro, Gabriella Giacon, Kushan Gandhi, Jeffrey Huang, Omar Mohyieldin, Alexander Hart, Mostafa Amer, Kate Dunstall, Tina Zhang, Ria George, Sang Ho Kim, Stevie Waerea, Kate Henley, Daniel Wen, Haru Yasutomi, Isaac Samuels, Ana Menon, Boaz Competente, Michael O’Grady, Thitapon Uiyapat, Mazoon Said Ahmed Al Jabri, Luis Bejarano Redondo, Cristina Lasa Unzue, Raquel Chaves Lopez, Beatriz Ledesma Muñoz, Irene Santos Prado, Maria Angeles Mancebo Zafra, Laura Hughes Lopez, Sahely Flores Risco, Daniel Marivela Palacios, Alvaro Martos Polo, Laura Soriano Melero, Hugo Eduardo Martin Acero Perez, Javier Feito Sancho, Raquel Torrillas Lopez, Guillermo Ocaña Ojea, Ana Isabel Vidal Conde, Irene Sastre Yague, Alvaro Revuelta Rebollo, Magali Ruiz Medrano, Elena Fayos Herrera, Raquel Munoz, Carrmen Ramon, Mercedes Cabellos, Coral Ramirez, Enrique Lopez Ruiz, Marta Ubre, Julia Martinez Ocon, Guillermo Laguna, Raquel Risco, Purificacion Matute, Manuel López-Baamonde, Marina Vendrell, Pinar De Santos, Beatriz Tena, Paula Masgoret, Guido Munoz, Juan Vidal Diaz, Lourdes Duran, Adriana Monente, Angela M De Molano, Brezo Del Blanco, Irene Murguialday, Juanjo Sanchez Palomo, Estrella Valencia, Blanca Carrillo, Marta Embid, Victoria Acedo, Nelia Tabatabaian, Ruben Sanchez, Pelayo Cadenas, Vlada Bizga, Alejandra Lasprilla, Carlos Aguilar, Rosa Beltrao, Tania Fernandez, Ana Mcg, Pedro Calle, Maria Jose Reyes, Kevin Romero, Guillermo Martin, Nacho Nunez, Luisa Bolivar, Jaime Rodriguez, Rocío Gutiérrez Bustillo, Julia Martín-Romo Capilla, Juan Manuel Priede Vimbela, María José Cobreces García, Álvaro Regueira Fernández, José Ignacio Andrés Viloria, Diego Palenzuela Blasco, Gema Rodríguez Cerón, Ángel Fernández Collantes, Rocío López Herrero, Lucia Gil Gomez, Jesus Fernanz Anton, Veronica Gonzalez Santin, Eva Bassas Parga, Natalia Montero Gaig, Jordi Castellvi Valls, Laura Grau Torradeflot, Luisa Fernanda Cueva Castro, Jordi Genoves Gonzalez, Patricia Calvo Pasaron, Carlos Jerico, Khadidjettou Mohamed Yahadih, Ana Tejedor Navarro, Marta Garcia Martinez, Alex Romero Fernández, Sainz Lete, David Gomez Dominguez, Maria Jesus Sanz De Leon, Rodrigo Urruchi, Laura Vaquero, Patricia Rodriguez, Manuel Carazo, Alba Perez, Alvaro Gudina, Maria Jose Blanco, Irene Arranz, Delia Velasco, David Martin, Elena Laita, Maria Garcia, Marta Ramos, Esther Aguado, Eugenio Ruiz De Santos, Laura Leal, Alicia Bordell, Cristina Barbosa, Ester Mora, Antonio Navarro, Guillermo Puig, Inma Camprubi, Marta Caballero, Adriana Rueda, Miguel Victoriano Solis Sanchez, Maria Huerta Carrascosa, Juan Pablo Nocete Navarro, Elena Murcia Sanchez, Jose Javier Herrero Parras, Paula Rodriguez Nieto, Isabel De La Calle Gil, Maria Gonzalez Cofrade, Rosalía Navarro Casado, Miguel Cendrero Martin, Adrian Martinez Lopez, Gabriela Seronero Vasquez, Alvaro Ramiro Ruiz, Pablo Garcia Pimentel, Maria Labalde Martinez, Cristina Nevado Garcia, Javier Garcia Borda, Pablo Pelaez Torres, Alfredo Vivas Lopez, Oscar Garcia Villar, Tamara Llamero, Alicia Ferrer, Marta Gonzalez Bocanegra, Alba Manuel Vázquez, Ignacio Aguila Collantes Velasco, Myrna Assaf Ballut, Nuria Baeza Pintado, Paloma Sanz Munoz, Ana Garcia Tello, Juan Boronat Catala, Juliusz Jan Szczesniewski, Jose Ramon March, Marina Garcia Cogollo, Cristina Hernandez Avila, Ainhoa Valle Rubio, Jose Luis Ramos Rodriguez, Alberto Hernandez Matias, Jose Mariá Jover Navalon, Mercedes Martinez Garcia, Celddy Mireya Urquizo Torrico, Silvia Benito Barbero, Justyna Drewniak Jakubowska, David Stolle Duenas, Ana Nieto Moreno, Alfredo Abad Gurumeta, Paula Fernandez Valdes Bango, Alicia Ruiz Escobar, Diana Fernandez, Javier Ripolles Melchor, Elena Saez Ruiz, Elena Nieto Moreno, Norma Aracil Escoda, Elena Lucena De Pablo, Marina De Retes, Kateri Chao, Berenice Gutierrez Tonal, Eugenio Martinez Hurtado, Mercedes Garcia Dominguez, Barbara Algar Yanez, Paloma Munoz Saldana, Ana Maria Tirado Errazquin, Sandra Maria Gadin Lopez, Rut Salvachua, Beatriz Nozal, Cristina Sevilla Guillen, Rosa Sanz Gonzalez, Olga De La Varga Martinez, Marina Valenzuela Pena, Beatriz Vazquez Rivero, Patricia Serrano-Mendez, Alexander Forero-Torres, Fuad Lopez-Fernandez, Francisco J Reinoso, Clara Marchesi Alcober, Begona Ayas Montero, Sandra Verdeguer Ribes, Maria Jose Alberola Estelles, Ana Navarro Barles, Xenia Carre Rubio, Ildefons Sarda Aure, Xenia Carre Rubio, Marta Silvestre Cepeda, Enrique Ruiz Velasquez, Sami Galal Eldin, Wael Alkhaleel, Hala Alhomi Alsaid Mushaweh, Ahmad Alhaj, Marwan Al Aliwy, Marah Zeidani, Abdallah Aladna, Kusay Ayoub, Lama Kadoura, Mousa Sifat, Joudi Chachati, Ammar Niazi, Ehab Alaasy Alkushti, Hilal Matar, Abdalwahab Alkhalaf, Opadah Salhab, Masa Watfa, Alhasan Alkhayer, Habib Olaisheh, Ali Hammed, Ahmad Molhem, Ali Sulaiman Deeb, Naya Hassan, Bassam Hassan, Ali Esmael, Hamza Ibrahim, Zuhoor Almohanady, Ghadeer Al Wajeeh, Khadega Abotaleb, Waheeb Al Kubati, Ghofran Al Nahwi, Ashwaq Ziyad, Hanan Sheebren, Faris Alhajami, Hanadi Ali, Wejdan Al Wafi, Amani Altaweel, Belquis Aljailani, Salwa Al Eryani, Abeer Mohammed, Abeer Saadan, Bushra Alshaikh, Shada Alemad, Manal Aldhaheri, Qasim Aldhaheri, Rafat Al Saban, Suhail Alshehab, Heba Abduljawad, Maha Muharram, Amal Muharram, Eman Al Markiz, Sarah Shream, Hanan Alhakimi, Shaima Ziyed, Ansam Alrobaiee Al Thawra

**Affiliations:** The University of Western Australia, 35 Stirling Highway, Perth WA 6009, Australia; Fiona Stanley Hospital, 11 Robin Warren Drive, Perth WA 6150, Australia

## Introduction

Preoperative anaemia is observed in one-third of patients who present for surgery, and is associated with increased postoperative complications, length of stay, poorer quality of life and delayed recovery^1,2^. In the last decade, there has been a focus on the detection and management of anaemia, prevention of bleeding and appropriate blood transfusion practices, collectively termed patient blood management (PBM)^3^. Implementation of PBM programmes is associated with reduced blood transfusion use and shorter hospital stay^4,5^.

In surgical patients, preoperative anaemia can be exacerbated by blood loss at the operation and repeated blood testing during and after the procedure. As iron deficiency is the commonest underlying cause of anaemia, the use of intravenous (IV) iron is a plausible and attractive therapeutic option to improve haemoglobin levels^6^. However, two recent large RCTs on the use of preoperative IV iron in major abdominal surgery, PREVENTT and FIT, did not show benefit in the immediate perioperative period on blood transfusion rates, complications or length of stay. However, an interesting finding in both trials was that the greatest treatment effect was increased haemoglobin levels at 6–8 weeks after surgery, which was associated with a reduction in unplanned hospital readmissions^7,8^. The association of postoperative anaemia and worse patient recovery was also observed in a large database analysis of patients undergoing surgery^9^. In a reanalysis of a large perioperative clinical trial, postoperative anaemia was associated with an increased risk of death and disability at 90 days^10^.

The mechanism of postoperative anaemia leading to delayed postoperative recovery is possibly due to the symptoms associated with the condition, including impaired mental function and reduced physical exercise capacity^11^. In the elderly, anaemia is associated with functional loss and increased risk for developing frailty^12–15^.

The prospective international POST-operative Variability in anaemia treatmenT and Transfusion (POSTVenTT) cohort study aimed to determine whether postoperative anaemia was associated with poorer recovery after surgery, with an increased rate of readmissions.

## Methods

The POSTVenTT study was a prospective, multicentre, international observational cohort study involving sites across Australia, Aotearoa New Zealand, Europe, North Africa and the Middle East. The POSTVenTT study was registered with the Australian New Zealand Clinical Trials Registry (ACTRN12621001517864; 8 November 2021). The trial protocol was approved by the South Metropolitan Health Service (Western Australia) Health Research Ethics Committee (EC00265), the Western Australia Research Governance System (RCG 4477), with local ethics and governance confirmed at each site before patient recruitment^16^. Sites were recruited utilizing collaborative research networks such as TASMAN and EuroSurg. This study is reported in concordance with the STROBE statement^17^.

### Study design

POSTVenTT was conducted by utilizing existing student- and trainee-led collaborative research models^18^. An advisory board of surgical trialists supported by the Western Australia Clinical Trials Unit oversaw a student- and trainee-led project management group. Data was collected over two 2-week periods during July 2021 at sites across Australia and Aotearoa New Zealand, and additionally in October 2021 at sites in Europe, North Africa and the Middle East. Each site was responsible for a 30-day prospective follow-up after discharge.

### Eligibility criteria

All consecutive adult patients aged 18 years or above undergoing major emergency or elective abdominal surgery were eligible. Major abdominal surgery was defined as any operation with an incision into the abdominal cavity (open, laparoscopic, or robotic surgery) and an anticipated duration of more than 1 h. Patients were recruited upon booking for an eligible procedure and upon procedure completion the operation report was reviewed for eligibility. Patients with planned readmissions were included due to the existing potential for unplanned readmission. Full details of eligible procedures and eligibility criteria are listed in the study protocol^16^.

### Definitions and outcomes

Anaemia was defined using the WHO sex-specific cut-off haemoglobin values (<130 g/l for men, <120 g/l for non-pregnant women)^19^. Mild anaemia was defined as a haemoglobin >100 g/l, and moderate to severe anaemia <100 g/l. The primary outcome was the rate of unplanned readmission to the hospital within 30 days after initial discharge. Secondary outcomes included clinical frailty score, length of postoperative hospital stay, postoperative complications (defined by the Clavien–Dindo classification), packed red blood cell (pRBC) transfusion and the prevalence of anaemia at discharge and 30 days post-discharge. Frailty was defined according to the CSHA Clinical Frailty score, and further classified as ‘Not Frail’ (1–3), ‘Mild Frailty’ (4–5), and ‘Moderate to Severe Frailty’ (≥6)^20,21^. Prolonged length of hospital stay was defined as a postoperative hospital admission greater than that of the 75th centile of patients in the study.

### Statistical analysis

R version 3.6.1 (R Foundation for Statistical Computing, Vienna, Austria) was used for all analyses. It was prespecified that data would only be included from hospitals with >95% data completeness as per the study protocol.^16^ Categorical data were presented as numbers and percentages, analysed using the chi-square test. Continuous data were presented as means with standard deviations or medians with interquartile ranges as appropriate and analysed using the independent samples *t*-test or Kruskal–Wallis test.

Time to readmission within 30 days from discharge was analysed as a time-to-event variable, using multivariable Cox regression models, reported as adjusted hazard ratios (aHR) with associated 95% confidence intervals). To analyse postoperative changes in frailty, this outcome was dichotomized as ‘more frail’ *versus* ‘unchanged/less frail’ at 30 days follow-up compared with preoperative frailty, and this was analysed using multivariate logistic regression models, reported with adjusted odds ratios (aOR) and 95% c.i. These models were adjusted for age, sex, ASA, cardiac history, diabetes mellitus, preoperative frailty, operative urgency, operative procedure, wound contamination, postoperative complications, postoperative pRBC transfusion and country of treatment.

Adjusted restricted cubic spline models were used to investigate the effect of haemoglobin at discharge as a continuous variable, for both readmission and frailty outcomes. For these models, splines were placed at the 5th, 25th, 75th and 95th percentiles. Multiple imputations with the mice (Multivariate Imputations by Chained Equations) package were used to generate 10 imputed data sets, and these were pooled to conduct the analyses described above. A sensitivity analysis was also performed with non-imputed data and pairwise exclusion of patients with missing data.

A post-hoc sensitivity analysis was performed excluding patients undergoing cholecystectomy, as although defined as major surgery, cholecystectomy is often performed as a day-case procedure and is associated with lower risks of bleeding and anaemia.

## Results

In total, 5266 patients undergoing major abdominal surgery at 101 hospitals across 13 countries, with a median of 47 patients (i.q.r. 21–77) per hospital were included, of whom 5143 (97.7%) were discharged before the end of the study. Data were complete for 99.3% of all data fields. Of those discharged prior to the end of the study, 30-day follow-up data were completed for 5050 patients (98.2%). Only patients with 30-day follow-up were included in the analysis. The mean age was 55.6 (s.d. 16.8) years, and 2944 (58.3%) were women (*Table 1*).

**Table 1:** Demographic and preoperative clinical data, by readmission within 30 days of discharge

	Readmission (%)	No readmission (%)	*P*
Total *N*	343 (6.8)	4707 (93.2)	
Mean age (years)	58.7 (16.7)	55.3 (16.8)	<0.001
**Sex**			
Male	187 (6.4)	2757 (93.6)	0.174
Female	155 (7.4)	1949 (92.6)	
Mean BMI (kg/m^2^)	29.2	29.5	0.549
**ASA physical status**			
1–2	181 (5.4)	3169 (94.6)	<0.001
3	137 (8.9)	1397 (91.1)	
4–5	23 (14.8)	132 (85.2)	
Cardiac disease	98 (8.5)	1055 (91.5)	0.012
Respiratory Disease	64 (8.0)	741 (92.0)	0.19
**Diabetes mellitus**			
Diet	8 (8.5)	86 (91.5)	0.07
Medication	45 (9.3)	438 (90.7)	
Insulin	15 (8.6)	159 (91.4)	
Neurological disease	25 (8.7)	262 (91.3)	0.235
**Liver disease**			
Mild	15 (11.7)	113 (88.3)	0.066
Moderate to severe	4 (9.3)	39 (90.7)	
**Smoking**			
Current	68 (7.9)	794 (92.1)	0.369
Previous	71 (6.5)	1027 (93.5)	

The most common operations were hepatopancreatobiliary (38.4%), colorectal (25.4%) or upper gastrointestinal (16.9%). Urgent or immediate surgery occurred in 853 patients (16.9%). Postoperative (in-hospital) complications occurred in 941 patients (18.6%), of which 203 (4.0%) were classified as major (Clavien–Dindo ≥3). Blood transfusion was used intraoperatively in 259 patients (5.1%) and postoperatively in 354 patients (7.0%) (*Table 2*).

**Table 2:** Operative clinical data, by readmission within 30 days of discharge

	Readmission (%)	No readmission (%)	Total	*P*
**Surgical specialty**				
Colorectal	126 (9.8)	1157 (90.2)	1283	<0.001
Gynaecology	28 (5.7)	459 (94.3)	487	
HPB	98 (5.0)	1843 (95.0)	1941	
Transplant	12 (26.1)	34 (73.9)	46	
Upper GI	48 (5.6)	806 (94.4)	854	
Urology	30 (7.7)	361 (92.3)	391	
Vascular	1 (2.1)	47 (97.9)	48	
**Operative urgency**				
Immediate	7 (9.0)	71 (91.0)	78	0.001
Urgent	77 (9.9)	698 (90.1)	775	
Expedited	53 (7.4)	662 (92.6)	715	
Elective	206 (5.9)	3272 (94.1)	3478	
**Operative contamination**				
Clean	122 (5.4)	2145 (94.6)	2267	0.001
Clean–contaminated	179 (7.7)	2141 (92.3)	2320	
Contaminated	29 (8.5)	314 (91.5)	343	
Dirty	12 (11.8)	90 (88.2)	102	
**Intraoperative allogenic PRBC Transfusion**				
No	308 (6.4)	4482 (93.6)	4790	<0.001
Yes	34 (13.1)	225 (86.9)	259	
**Postoperative ICU admission**				
No	264 (6.4)	3873 (93.6)	4137	0.012
Yes—planned	66 (8.2)	743 (91.8)	809	
Yes—unplanned	13 (12.5)	91 (87.5)	104	

Overall, the median length of hospital stay was 3 days (i.q.r. 1–6 days). Haemoglobin values were measured for 3672 patients (72.7%) postoperatively, with a mean haemoglobin at discharge of 116.8 g/l (s.d. 17.4 g/l). Anaemia was common on discharge; 2340 patients (46.3%) of whom 1698 (33.6%) had mild anaemia (Hb > 100 g/l) and 642 patients (12.7%) moderate–severe anaemia (Hb < 100 g/l). Among patients with anaemia at discharge, 709 (30.3%) had a follow-up haemoglobin measurement. Of those who had follow-up haemoglobin measurements, 501 (70.7%) had anaemia at 30 days (*Table 3*).

**Table 3:** Postoperative clinical data, by readmission within 30 days of discharge

	Readmission (%)	No readmission (%)	Total	*P*
**Postoperative complications**				
None	225 (5.5)	3883 (94.5)	4108	<0.001
Minor	82 (11.1)	656 (88.9)	738	
Major	36 (17.7)	167 (82.3)	203	
Postoperative PRBC transfusions	56 (13.9)	355 (7.3)	411	<0.001
**Reoperation**				
No	330 (6.7)	4610 (93.3)	4940	0.05
Yes	13 (11.9)	96 (88.1)	109	
**Length of stay**				
Mean(s.d.)	7.7(8.3)	5.2(6.8)	5.3 (7.0)	<0.001
**Anaemia at Discharge**				
No Anaemia	73 (5.5)	1259 (94.5)	1332	<0.001
Anaemia	223 (9.5)	2117 (90.5)	2340	
**Haemoglobin at discharge**				
Mean(s.d.)	110.9(17.5)	117.3(17.3)	116.8 (17.4)	<0.001
**Postoperative iron therapy**				
None	306 (6.5)	4410 (93.5)	4716	<0.001
Oral iron	12 (6.8)	164 (93.2)	176	
IV iron	25 (16.1)	130 (83.9)	155	

### Readmission within 30-days of discharge

Within 30 days of discharge 343 patients (6.8%) were readmitted to hospital, with a median time to readmission of 10 days (i.q.r. 5–18 days). Of the patients readmitted, 314 (91.5%) were unplanned readmissions. The most common reasons for readmission were postoperative surgical complications, general infections and wound infections, 45.5%, 16.6%, and 14.3% respectively (*Table S1*).

**Fig. 1:**
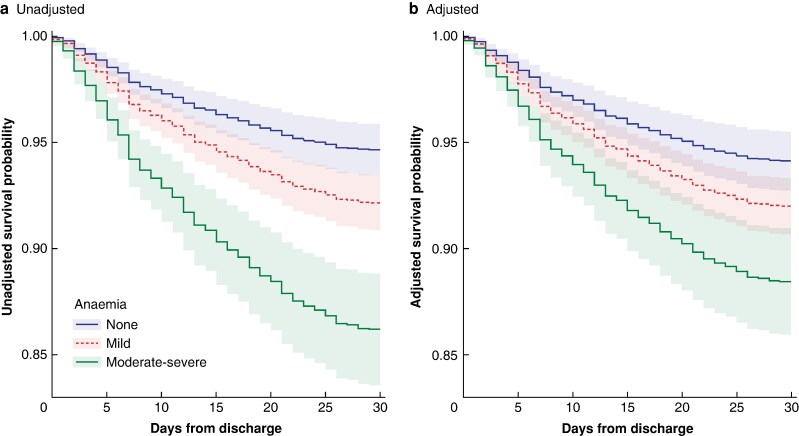
a Unadjusted and b adjusted Cox regression curves for readmission-free survival for patients discharged without anaemia, and with mild or moderate–severe anaemia

**Fig. 2:**
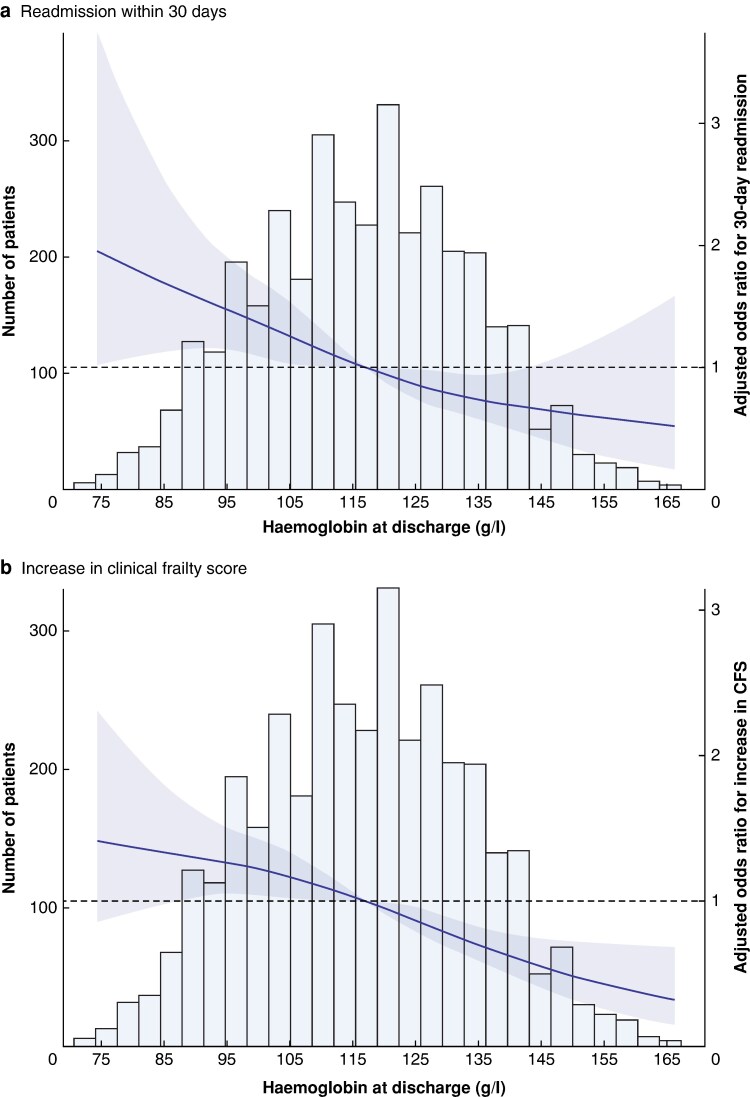
Restricted cubic spline regression for the association between haemoglobin at discharge and a adjusted odds for readmission within 30 days of discharge, and b adjusted odds for increase in frailty at 30-day follow-up Knots are placed at percentiles 5, 25, 75, 95; Hb = 88, 104, 129, 145 g/l. CFS, clinical frailty score.

Patients with postoperative anaemia were more likely to be readmitted within 30 days, 223/2340, 9.5% *versus* 73/1332, 5.5% patients without anaemia, *P* < 0.001. Both unadjusted and adjusted Cox regression models confirmed postoperative anaemia was associated with an increased risk of readmission (unadjusted HR 1.80, 95% c.i. 1.38 to 2.36, *P* < 0.001, and aHR 1.56, 95% c.i. 1.18 to 2.08, *P* = 0.002; *Table 3*). Moreover, a severity-dependent effect was seen; the more severe the anaemia, the greater the associated risk of readmission: moderate–severe postoperative anaemia (aHR 2.08, 95% c.i. 1.46 to 2.96, *P* < 0.001) and mild anaemia (aHR 1.41, 95% c.i. 1.04 to 1.90, *P* = 0.03) compared to patients without anaemia (*Fig. 1*). Adjusted restricted cubic spline regression demonstrated higher adjusted odds of readmission associated with lower haemoglobin values at discharge from the hospital (*Fig. 2a*).

### Frailty

Clinical frailty scores were recorded for 99.3% of patients before surgery, and 95.9% of patients at 30-day follow-up. Preoperatively, 651 patients (12.9%) were classified as having mild frailty and 129 (2.6%) had moderate or severe frailty. Preoperative frailty was associated with an increased risk of postoperative complications (aOR 1.26, 95% c.i. 1.01 to 1.57, *P* = 0.049), prolonged length of stay (aOR 1.28, 95% c.i. 1.00 to 1.63, *P* = 0.048) and readmission within 30 days of discharge (aHR 1.41, 95% c.i. 1.05 to 1.88, *P* = 0.02). At 30-day follow-up, 867 patients (17.2%) reported increased frailty, with a mean increase in the clinical frailty score of 1.3 points (s.d. 0.7) in this group (*Table S2*).

Patients discharged with postoperative anaemia were more likely to report an increase in frailty at 30-day follow-up (24.9% anaemic *versus* 14.8% not anaemic; aOR 1.64, 95% c.i. 1.35 to 2.01, *P* < 0.001). The severity of anaemia was associated with a greater risk of worsening clinical frailty score; moderate–severe anaemia (aOR 2.05, 95% c.i. 1.44 to 2.91, *P* < 0.001), and mild anaemia (aOR 1.36, 95% c.i. 1.01 to 1.85, *P* = 0.04) at discharge were both associated with increasing frailty scores at 30-day follow-up. Restricted cubic spline analysis also showed lower haemoglobin values at discharge were associated with higher odds of increased frailty at 30-day follow-up (*Fig. 2b*).

### Sensitivity analyses

Sensitivity analyses performed using pairwise exclusion of missing data rather than multiple imputations showed no meaningful differences compared to the main analysis. A subgroup analysis excluding patients undergoing laparoscopic cholecystectomy was also performed (*n* = 3323). Postoperative anaemia was associated with an increased risk of readmission (aHR 1.68, 95% c.i. 1.19 to 2.38, *P* = 0.004), and an increase in frailty at 30-day follow-up (aOR 1.57, 95% c.i. 1.24 to 1.99, *P* < 0.001). The severity of anaemia increased the risk of both readmission (mild aHR 1.52, 95% c.i. 1.06 to 2.19, *P* = 0.02; moderate–severe aHR 2.11, 95% c.i. 1.41 to 3.18, *P* < 0.001) and becoming more frail at 30-day follow-up (mild aOR 1.49, 95% c.i. 1.17 to 1.90, *P* = 0.001; moderate–severe aOR 1.82, 95% c.i. 1.35 to 2.45, *P* < 0.001).

## Discussion

In this international prospective study (POSTVenTT), anaemia after major surgery was common and had a negative impact on patient recovery, with an increased risk of unplanned readmission and increased frailty at 30 days. These results confirm the findings of previous retrospective database analyses and build on the secondary findings seen in the PREVENTT and FIT RCTs, including the reanalysis of trials which suggested a greater risk of mortality or disability up to 90 days following surgery, postoperative delirium and a longer length of stay^9,10,22^.

Postoperative anaemia is more common when there is pre-existing anaemia, significant blood loss, frequent blood sampling, excessive intravenous fluid administration, inflammation and nutritional deficiencies before or after surgery^23^. PBM strategies, such as a short-term administration of intravenous iron especially in the postoperative setting, should be considered for patients with moderate-to-severe iron deficiency to address postoperative anaemia^24^. Also, tranexamic acid use has been consistently reported to be effective in the perioperative period, but it is still under-utilized^25,26^.

Current recommendations propose the use of IV iron to treat patients with anaemia, ideally 4 weeks before their planned surgery^27^. However, this may not be feasible in the timelines before abdominal surgery with most clinical trials recruiting about 2 weeks before operation^28^. There is debate about the efficacy of IV iron to increase haemoglobin levels before surgery, particularly as it is not clear about the impact of inflammation and chronic disease on iron transport and sequestration. Although absolute iron deficiency would mandate treatment in any hospitalized patients with ferritin < 30 μg/l, the proposal for treatment in patients with ferritin < 100 μg/l or transferrin saturations < 20% may be less effective^29^. This in part may explain why IV iron may be more beneficial in patients undergoing bowel surgery, where the cause of anaemia is likely gastrointestinal blood loss^30^. As such, administration of IV iron after surgery where anaemia may be caused by blood loss at operation and in the postoperative period may have a more direct impact on iron stores that may override the inflammatory mediated iron sequestration. Similarly, administration of IV iron after surgery, when the patient is on the ward, with IV access and monitoring, would be easier and less expensive than intravenous iron administered at preoperative visits^26,31^.

The present study has demonstrated an association between anaemia at discharge and increasing frailty at 30 days post-surgery (aOR 1.65, *P* < 0.001). This highlights the importance of evaluating frailty in anaemic individuals and investigating any potential negative consequences of the co-existence of these two conditions. Previous research supports the relationship between anaemia and frailty^31,32^. The connection between anaemia and frailty may be due to the requirement for iron by cellular and mitochondrial metabolism and its impact on skeletal muscle function, leading to sarcopenia and myopathy^33^. These findings have clinical implications as they highlight the need to evaluate frailty in anaemic individuals.

A significant strength of the study was the diversity of the study population from various countries in different geographical regionals (Australia and Aotearoa New Zealand, Europe, North Africa and the Middle East) and economic status, with a high (98%) degree of data ascertainment. The results should therefore be reliable and readily translated globally.

The POSTVenTT study has several limitations. The study design was observational, which only allows for the identification of correlations between anaemia and postoperative outcomes, limiting the causal inferences that can be made. Additionally, the measurement of haemoglobin values was incomplete, covering only 72.7% of patients postoperatively, which may affect the generalizability of the results. This observational study was susceptible to variable sampling bias in the different specialties and hospitals, which may have influenced the apparent variation in adherence to guidelines. Furthermore, as the clinical frailty score was collected preoperatively and at 30 days after surgery, further research is required to determine if the impact on frailty is longer lasting. Patients who underwent staged procedures with the index surgery prior to the recruitment period but subsequent surgery fell within the recruitment period were included in the study. Unfortunately, data was not collected to quantify the number of patients who met this criteria. These patients would understandably be at higher risk for readmission and complications, and thus a potential unquantified confounder of the data set.

A further limitation is the range of procedures included and by association the range of primary pathology treated. The primary pathology often dictates the morbidity of patients and residual confounding could have played a role in the outcomes seen in this study. However, to address this potential limitation, a sensitivity analysis was performed together with reanalysis of the data without including patients undergoing laparoscopic cholecystectomy (the most common procedure performed). This analysis had the same findings as the whole study population.

Future research should build on the POSTVenTT study through RCTs that aggressively correct perioperative anaemia prior to discharge to determine if anaemia alone causes an increased rate of readmission, rather than act as a marker of co-morbidity.

## Collaborators

Kenneth J. Macpherson, Ane Abad Motos, Vinicio Mosca, Ruth Blanco Colino, Muhammed Elhadi, Eman Ali, Cameron Wells, Chris Varghese, William Xu, Jurstine Daruwalla, Gianluca Pellino, Jose Antonio Garcia Erce, Nagendra N. Dudi-Venkata, Toby Richards, David I. Watson, Pete Pockney, Deborah M. Wright, Doug Robb, Guiliana D’Aulerio, Jani Lee Moss, Uyen G. Vo, Jai Darvall, Matthew A. Warner, Warren Seow, Laure Taher Mansour, Jayne Lim, Su Kah Goh, David S. Liu, Muhammad Ibrahim, Amanda C. Dawson, Elizabeth W.Y. Lun, Adam J. Frankel, Siang Wei Gan, Jonathan Foo, Francesco Pata, Liam Ferguson, Daniel Chubb, Christophe Berney, Sonalmeet Nagra, Chonhann Liew, Hasanga Jayasekera, Merwe Hartslief, Adeep Majid, Henry Y. Cheung, Sayed Hassen, John VanBockxmeer, Tobias J. Evans, Francesco Amico, Girish Pande, Justin S. Gundara, Elizabeth Murphy, Bernd Froessler, Sarah Martin, Daniel Croagh, William Teoh, Matthias Wichmann, Jonathan Fong, Krinalkumar Mori, Quentin Ralph, Aliakbar Estakhri, Sarah R. Bowman, Harsh Kanhere, Benjamin Loveday, Stephanie Chetrit, Dale Currigan, Mary Theophilus, Michael J. Johnston, Markus Trochsler, Justin Yeung, Guy Maddern, Richard G. McGee, Mohamed Asal, Mostafa Abbas, Giuseppe Spampinato, Donato F. Altomare, Piergiorgio Danelli, Andrea Mingoli, Simone Giacopuzzi, Luca A. Fumagalli, Stefano Olmi, Marzia Franceschilli, Adolfo Pisanu, Ghaith Bani Abdel Rahman, Milad Gahwagi, Ian Bissett, Jeremy Rossaak, Saxon Connor, Alexander W Brown, Andrew MacCormick, Sze-Lin Peng, Peter Stiven, Bevan Jenkins, James Tietjens, Christopher Harmston, Christopher M. Gray, James G. Wilkins, Jasen Ly, Andrew Herd, Sarah Rennie, Marianne Lill, Raed Amro, Jose Ramon Rodriguez Fraile, Maria Moral Gonzalez, Santiago Valderrabano-Gonzalez, Montasir A. Abdalbain, Ahmad Ghazal, Nashwan Tashan, Mohammed Al Shehari, Jed Hughes, Joseph C. Kong, Melanie E. Cusso, Daniel R. Cox, Sarit S. Badiani, Sara Mohammed Jinnaah, Amy Crowe, Enoch Wong, Melissa Y. Wee, Matthew J. Marino, Isabella Ludbrook, Anne-Marie Aubin, Timothy J. Ganguly, Tara Kannan, Fiona Pavan, Geraldine J. Ooi, Vignesh Narasimhan, Paul T. Heitmann, Tiffany J. Cherry, Sharon Lee, Teng-Wei Khoo, William Lindores, Christine Li, Xiao-Ming S. Woon-Shoo-Tong, Amelia L. Davis, WeiShearn Poh, Thomas Tiang, Victor W. Chai, Gayatri Asokan, Eunice Tse, Brianne Lauritz, Alicia Lim, Mahmoud Shaban Abdeljalil, Moataz Ewedah, Ahmed Abbas, Marian Abou El Garad, Hossam Elfeki, Mohamed Elbahnasawy, Stefano Garritano, Arcangelo Picciariello, Francesco Colombo, Pierfrancesco Lapolla, Paola De Nardi, Mauro Zago, Matteo Uccelli, Giuseppe Sica, Mauro Podda, Almu’atasim Khamees,, Wafa Aldressi, Eman Abdulwahed, Jelle M. Vander Have, Imogen I. Watt, Brodie M. Elliott, Chelsea A. Allen-Brough, Carys Finlayson, Olga Korduke, Mathew Morreau, George N. Weeratunga, Matthew J. McGuinness, Jethro Palmer, David C. Kieser, Xavier Field, Dhenisha Dahya, William Xu, Louis-Antoine Bonnet, John George Grace Massoud, Sarah Amro, Fátima Cañeque Yanini, Beatriz Amoros, Aranzazu Calero Lillo, Misericordia Basora, Rosalia Navarro Perez, Rita Pilar Rodriguez Jimenez, Carmen Deiros Garcia, Aitor, Cesar Aldecoa, Montse Mallol Oliva, Raquel Garcia Alvarez, Virginia Jiménez Carneros, Raquel Fernandez Garcia, Hanna Perez-Chrzanowska, Maria Vila Montanes, Maria Luisa Pinana Campon, Hytham K.S. Hamid, Ruqaya Masri, Abdulrahman Almjersah, Ali Alloush, Fatima Al Eryani, Jonathon Holt, Stephanie Potenza, Avelyn Wong, Sagarika Handa, Virginia Su, Aniqa Hussain, Angus Tolhurst-McKiernan, Amelia J. Cooper, Mark T. Butorac, Hugh Elbourne, Emily Dutta-Powell, Neave Kapoor, Caitlin Reid, Jason Wang, Stella Le, Jordi Elliott, Bill Wang, Jiting Li, Zhuoning Song, Aya Basam, Branavan Thevashangar, Shani Nguyen, Arsheeya Rattan, Jeffrey Shao, Cynthia Wells, Emma Aldous, Siobhan Brodrick, Brooke Callahan, Faizah Alam, Laura Casey, Wilson Yang, Swetha Prabhakaran, Haini Jiang, Alex Lin, Angela Chau, Pranjal Chaurasia, Morgan Jones, Yit J. Leang, Wendy Brown, Jonathan Serpell, Peter Carne, Matthew Stokes, Jern Cabral, Miriam W Bennamoun, Suneha Lal, Kai Dong Choong, Yanran Yu, Chris Zhao, Vijayaragavan Muralidharan, Sean Stevens, Liam Fernando-Canavan, QiRui Soh, Nakjun Sung, Grace Gold, Alex Grogan, James G. Lin, Ahmad Aly, Georgina Riddiough, Hamza Ashraf, Michaela Wojno, Adrian Yeoh, Gabriel Wall, Jack Liu, Sonia Gill, Yida Zhou, Robert Torode, Michael Issa, Jasun Li, Ruben Schuringa, Marcos V. Perini, David Proud, Kevin Jia, Suhasini Shanmugarajan, Gladys Tam, Ashley Wang, Shipra Sankpal, Matthew Heard, Yung-Hsin Hsueh, Scott Anderson, Vishwakar Panuganti, Zoe Zhang, Gabriel Lirios, Joshua Birnie, Benjamin Scott, Lei Ying Barwon, Laura LeHua He, Samantha Mitchell, Chloe Wu, Brett Larner, Jason Fu, Amy McCormick, Daryn Goodwin, Alistair Lau, William Stockley, Ripudaman Jalota, Hannah Gora, Parvathy Ananthan, Thomas Estephan, Aakash Nanda, Sarika Suresh, Thihoai Trang Nguyen, Sarah McLain, Sharanya D. Mohan, Nicole Batten, Gajanth Sivapalan, Grace S. Ting, David Yoon, Joohyen Ahn, Lisa Shi, Salena Ward, Felicity Huang, Boya MacKeddie, Adrian Fox, JiYue Wang, Amie Hilder, Ashan Kathriachchige, Chen Lew, King Tung Cheung, Sarah Goh, Balakavitha Balaravi, Simran Vinod Benyani, Chris Ip, Shomik Sengupta, Christopher Steen, Janindu Goonawardena, Shantanu Joglekar, Erica Wang, Olivia Lin, Michelle Yao, Vinna An, Connor Burley, Raaj Chandra, Anshini Jain, Amelia Davies, Matthew Mann, Anand Trivedi, Matthew J Pugliese, Ron Ramos, Davina Daudu, Tasvinder Hans, Audrey Kim, Aiden Stuart, Harvinder Hans, Natalie Layden, Pravin Viswambaram, Ashley Lee, Mohammed Al-Zubaidi, Dickon Hayne, Hayley J. McMillan, Jason D’Silva, Ben P. Finlay, Rebecca Fong, Penelope Peres, Gavin Nair, Suchitra KrishnanPillai, Yong Zhi Beh, Steven L. Due, Benjamin Howes, Dominic Parker, Christina Mcveay, Matthew Marshall-Webb, Izhar-Ul Haque, AbdulAhad Rana, Sneha E. Ninan, Gemma J. Qian, Joseph W. Fletcher, Brendan Watkins, Luke C. Green, Yara A. Elgindy, Elodie V. Honore, Charles J. Roth, Jonathan Tandjung, Adam R. Perkovic, Luca Borruso, Victor Yu, Thomas-Hoang Le, Samuel M. Mathias, Daniel Jeong, Charlotte E. Cornwell, Emily A. Taylor, Kie Ren Oon, Mikayla Everuss, Jesse Hafner, Caleb Kim, Catherine T. Cartwright, Aram Cox, Ricci Amolis, Hui Kang Tan, Brittany Harrop, Theepiga Ramachandran, Elli Farrell, Saxon Farquhar, Felicity Goodes, Sophie Zhong, Sophie Kelly, Howard Li, Jun Jie Chau, Joshua Richards, Christopher Cheong, Tedman Chau, James Pankhurst, Darren J. Rama, Alicia Liong, Sebastian Primrose, Peita M. Webb, Edward Young, Harleen Kaur, Xuanyu Zhou, Mohamed Afzal, Jess Barnes, Allan Butler, Priya Nandoskar, Matt Besley, Mozafer Rajabali, Nikhil Kundu, Alexander Buckby, Linda Sunny, Olivia Anderson, Hemachalam Subramaniam, Sandeep Dayaratne, Anjelee Segaran, Daniel Chilton, Chris J. Kim, Dion Koh, Tom A. Eldredge, Syeda T. Saief, Kevin K. Yoong, Ashray Rajagopalan, Kathleen Wisbey, Chelsea Lin, Jolene Ong, Sandra Li, Jonathan Tiong, Naman Kohli, Joseph Jaya, Jiakai Yao, Evie Yeap, Katherine Grant, Genevieve Heath-Kalgutkar, Ryan R. Gosavi, David Zula, Ning Yih Kam, Travis Ackermann, Darryl Lee, Sean Lim, Alice Lee, Phi Nguyen, Lily Bae, Roshini Nadaraja, David Duong, Emma Payne, Natalie Nguyen, Gabrielle Cutler, Fellicia Stanzah, Matthew Watson, Luke Green, Aveechal Prasad, Laxmi Sistla, Emily Overton, Monika A. Wojt, Amos Moody, William Zore, Rebecca Blundell, Tarik Sammour, Syeda Khadijah Ahmed, Yickho Lam, EuLing Neo, Anthony Farfus, Leigh Warren, Zachary Bunjo, Jaspreet K. Sandhu, Qiwen Wang, Syeda Khadijah Ahmed, Nimantha Abey, Victoria Lu, Amanda Hii, Shalvin Prasad, Christopher Dobbins, Mohammad Mangi, Joseph Dawson, Danielle Boniface, Mathew Amprayil, Patwinder Gill, Jonathan McCafferty, S.F. Michelle Ku, Zijiang Yang, Ming Z. How, Frank Du, Reece Ansaar, Chelsea Xu, Nyan S. Lwin, Supisara Suk-Udom, Nathan Scott, Kyle Raubenheimer, Nivedan Jeyamanoharan, Priyanka Chakraborty, Bryce Stewart, Dinethma Tharuni Ekanayake, Diana Xiao, Aiswarya Ajith, Mark Ibraheem, Joseph Hanna, James Dodd, Sophie Zhao, Aine O’Dwyer, Sinali Seneviratne, Lauren Masi, Hilary Leeson, Xiao-Ming S. Woon-Shoo-Tong, Ho-Cing Yau, Daniel Lee, Siavash Mortazavi, Stephanie Bartley, Zheng DaChok, Nur Sabrina Binti Babe Azaman, Leesha Bryan, Bethany Furfaro, Peng Sheng Tan, Talia Shepherd, Mary Teoh, Kelly Shepherd, Danielle Malatzky, Nicole Hew, Pia J. Schelfhout, Rama Chidambaram, Ashwita S. Vanga, Madiha K. Bajwa, Aidan D. YueOye, Darren Karadimos, Nyssa Jualim, Vivien Hsu, Sharie A. Apikotoa, Cathy Maher, Emalee Burrows, Natalie Yu, Michael W. Hii, Lillian Taylor, Lynn Chong, Louis J. Sisk, Cathy Hua, Elaine Su, Aidan Jackson, Elizabeth Hu, Tori Wong, Andrew Xu, Toni Zhang, Luke Bradshaw, Kathleen Nagle, Antonio Barbaro, Lata Limbu, Mai Nguyen, Kalyanasundaram, Colin Choi, Ha M. Nguyen, Shyamolie Mathur, Nicholas Quirk, Carol Z. Wu, Reshi Suthakaran, Chui Foong Ong, Ali Baker, Rosalina Lin, Jade T. Bahnisch, Haili Luo, Mohamed Qassem, Omar Saeed Anas, Omar Abdelnasser Mohamed, Basil Alshami, Mariam Kefah Fathi, Muhammed Ahmed Muhammed Abdelrazeq, Hajar Fawzy Abdel Rady, Mahmoud El Garhy, Laila Raafat Mabrouk, Balqees Alhariri, Ahmed K. Awad, Merihan A. Elbadawy, Mahmoud Elsayed Abdelazeem Bakheet, Nadien Mohamed Abd El Hamid, Noha Eslam Attia Sakr, Yara Mohamed Yasser, Mostafa Osama Farag, Selwan Mahmoud Abdelrahman, Israa Mohamed Iraqui, Dalia Shaban Abdelgalil, Sherein Diab, Peter Nader Sobhi Rafla, Steven Danial Azmy Habib, Aya Abdulilah Abdulmonem, Bassant Hassan Shawki, Salma Adel Mohamed, Sally Ahmed Abd El Razik, Ahmed Hassan A. Rady, Ayatallah Ali Hussein Ramadan, Ayman K. Awad, Pavly Wagih Beshay, F. R. S. Gad, Salma Tarek Helmy, Michael Amin, Mahmoud Mohamed Mohamed Shalaby, Abanoub Ashraf Moufid Riyad, Ahmed Nafea, Marina Hany, Ahmed Sakr, Mohamed Al Sayed, Mosaab Tayiawi, Mohammed Eleleigy, Osama Elzankaly, Mohamed Mourad, Shaher Shokralla, Yomna Dean, Yousef Tanas, Samar Abdelnaby, Abdulrhman Abdulhameed, Afnan Morad, Esraa Emad Abdelazeem, Manal Abdelghany, Yosra Rateb, Eithar Alqady, Shereen Elnokrashy, Mona Helbawi, Asmaa Shaltout, Ahmed Hussein, Amira Salem, Ramy Hassan, Magdy Mahdy, Marina Adly, Sarah Abdelmoneim, Ahmed Taha, Ashraf Elnaggar, Mohamed Elkady, Omar Soliman, Ahmed Aboelmagd, Mahmoud Shehata, Shymaa Abodahab, Rehab Mohamed, Ahmed Youssef, Radwan Torky, Mohamed Abulfetouh, Gihan Galal, Sara Kalaifa, Mahmoud Khalil, Islam Ibrahim, Ahmed Kamel Morsi, Ahmed Wahba, Islam Abo Deef, Ahmed Abd Elzaher, Alaa Sultan, Abdallah Rashad Temerik, Ahmed Abdelmoneim, Shereen Saad, Shaymaa Ibrahim, Ahmed Elkoussy, Yasmine Adel Mohammed, Roaa Saleh, Randa El Zohne, Mohamed Shaker, Fatma Gharib, Esraa Kotb, Eman Hassan, Islam Aziz, Mohamed A. Abdelmaksoud, Ahmed Ramadan, Reem Elsaadany, Mohamed Taman, Karim Elkenawi, Esraa Daif, Hossam Elsonny, Rahma Elboraei, Mahmoud Laymon, Osama Ezzat, Ahmed Elbatta, Mohamed Hassan, Amr A. Elsawy, Ahmed Elsheshtawy, Islam Mansour, Amgad Fouad, Amr Sanad, Amr Kassem, Ahmed Beah, Ahmed Shehta, Ahmed E. Nabih, Rana Nagi, Mostafa Shalaby, Ahmed Sakr, Yousef Hesham, Sameh Emile, Aly Sanad, Ahmed Elsayed, Mirna Sadek, Omar Taha, Shady Elsalhawy, Mustafa Zaghloul, Olli Helminen, Heikki Huhta, Matti Aleksi Mosorin, Henri Juhani Sova, Ville Virta, Norma Depalma, Gabriella Lionetto, Stefano D’Ugo, Tiziana Marchese, Farshad Manoochehri, Federico Perrone, Rigers Dibra, Giuseppe Trigiante, Gennaro Martines, Luciano Rella, Marco Vulpi, Pasquale Ditonno, Giuseppe, Francesco Cammarata, Luca Ferrario, Andrea Bondurri, Alessandro Michele Bonomi, Anna Maffioli, Isabella Pezzoli, Albert Troci, Franco Palmisano, Andrea Gregori, Giacomo Piero Incarbone, Giancarlo D’Ambrosio, Andrea Picchetto, Elena De Stefani, Gioia Brachini, Flavia Ciccarone, Ilaria Clementi, Giuseppe Marenga, Piero Bartolucci, Gennaro Mazzarella, Bruno Cirillo, Immacolata Iannone, Cristina De Padua, Enrico Fiori, Paolo Sapeinza, Simona Meneghini, Martina Zambon, Giulia Duranti, Costantino Leonardo, Giorgio Franco, Pierfranco Maria Cicerchia, Luigi Simonelli, Giovanni Battista Fonsi, Sara Giovampietro, Edoardo De Meis, Monica Mansi, Giovanni Casella, Lidia Castagneto Gissey, Anna Falasca, Catia Gugliuccello, Barbara Binda, Maura Rocchetti, Sara Santillo, Federica Scarno, Andrea Vignali, Simonetta Massaron, Marco Ee Chiarelli, Fulvio Tagliabue, Alessia Malagnino, Giovanni Pesenti, Carmine Sciorio, Alessandro Maletta, Michele Morelli, Antonio Pellegrino, Claudio Reato, Giani Sofia, Giusi Giulia La Manna, Andrea Martina Guida, Bruno Sensi, Vittoria Bellato, Giuseppe Tisone, Roberta Angelico, Tommaso Maria Manzia, Leandro Siragusa, Giuseppe Farullo, Luca Orecchia, Lorenzo Alteri, Virgilio Michael Ambrosi Grappelli, Tiziana Pilia, Valentina Murzi, Gianmarco D ancona, Stefano Angioni, Monica Ortenzi, Maurizio Nicola D’Alterio, Alessandro Cois, Marcello Pisano, Luai Abu Ismail, Younis Maher, Abdallah Yasin, Aseel Almohtasib, Farah Al Kasaji, Saja Aloran, Lana Sbitan, Heba Abu Khalaf, Subhi Al Issawi, Radi Tofaha Alhusseini, Sajeda Awadi, Issa A. Abuzeid, Sarah Aldressi, Mohammed Alabeedi, Mohammed Moftah, Eman Salem, Mohammed N. Albaraesi, Haitham Elmehdawi, Ahmed Ahmayda, Ala Burgeia, Tarik Eldarat, Maruwan Safi Abdullah, Abraheem Aljibali, Rema Benhariz, Almotasem Bellah Elsharif, Yasmin Abu Ihlayqah, Intesar Elmejrisi, Fatimah Abouthiheer, Rabha Ibrahim, Ayoub N. Abdalmaged Akwaisah, Suha Younis Hasan, Ehsan Al Mahdi, Ehtuish Farag Ehtuish, Mohamed Alsori, Sultan Ahmeed, Sanad Kanna, Entisar Alshareea, Enas Soula, Sarah Aljamal, Ehda Kerwash, Doaa Gidiem, Ekram Abujrad, Hayat Ben Hasan, Aamal Askar, Esmail Hamuda, Khawla Derwish, Reem Ghmagh, Abdulqudus Deeknah, Fras Elhajdawe, Marwa Sinan, Jim Hsu-Shun Wang, Binura Lekamalage, Arpita Bansal, Luke DeRuiter, Kevin Yi, Faseeh Zaidi, Cindy Xu, Cain Anderson, Gabrielle Murti, Natasha P. Mitchell, Celine Peng, Olivia Mackay, Daniel A. Carson, Sameer Bhat, Jenny Choi, Justin Roake, Oliver Lyons, Eric T. Lim, MeiSze Lee, Jared McLauchlan, Claire Whitehead, Sandra J. Campbell, Fiona Carey, Grace Gatenby, Angharad Jones, Tamara Glyn, Heidi Blackburne, Emilie Roberts, Lauren Siggins, Renato Pitesa, Braedon Epps, Emma Espiner, Jack Hanlon, Ben Waller, Abhinav Bhasin, Sharmaine Sreedhar, Lauren Hubbard, Emily Evison, Molly Reynolds, Harriet Marshall, Sam Guest, Hinerangi Temara, Alexa Kuffel, Wing Yung Agnes Chu, Rebecca Teague, Rowan Auchterlonie, Vilingatoni Hasiata, Benjamin Keren, Joshua Tiro, Gabriella Giacon, Kushan Gandhi, Jeffrey Huang, Omar Mohyieldin, Alexander Hart, Mostafa Amer, Kate Dunstall, Tina Zhang, Ria George, Sang Ho Kim, Stevie Waerea, Kate Henley, Daniel Wen, Haru Yasutomi, Isaac Samuels, Ana Menon, Boaz Competente, Michael O’Grady, Thitapon Uiyapat, Mazoon Said Ahmed Al Jabri, Luis Bejarano Redondo, Cristina Lasa Unzue, Raquel Chaves Lopez, Beatriz Ledesma Muñoz, Irene Santos Prado, Maria Angeles Mancebo Zafra, Laura Hughes Lopez, Sahely Flores Risco, Daniel Marivela Palacios, Alvaro Martos Polo, Laura Soriano Melero, Hugo Eduardo Martin Acero Perez, Javier Feito Sancho, Raquel Torrillas Lopez, Guillermo Ocaña Ojea, Ana Isabel Vidal Conde, Irene Sastre Yague, Alvaro Revuelta Rebollo, Magali Ruiz Medrano, Elena Fayos Herrera, Raquel Munoz, Carrmen Ramon, Mercedes Cabellos, Coral Ramirez, Enrique Lopez Ruiz, Marta Ubre, Julia Martinez Ocon, Guillermo Laguna, Raquel Risco, Purificacion Matute, Manuel López-Baamonde, Marina Vendrell, Pinar De Santos, Beatriz Tena, Paula Masgoret, Guido Munoz, Juan Vidal Diaz, Lourdes Duran, Adriana Monente, Angela M. De Molano, Brezo Del Blanco, Irene Murguialday, Juanjo Sanchez Palomo, Estrella Valencia, Blanca Carrillo, Marta Embid, Victoria Acedo, Nelia Tabatabaian, Ruben Sanchez, Pelayo Cadenas, Vlada Bizga, Alejandra Lasprilla, Carlos Aguilar, Rosa Beltrao, Tania Fernandez, Ana Mcg, Pedro Calle, Maria Jose Reyes, Kevin Romero, Guillermo Martin, Nacho Nunez, Luisa Bolivar, Jaime Rodriguez, Rocío Gutiérrez Bustillo, Julia Martín-Romo Capilla, Juan Manuel Priede Vimbela, María José Cobreces García, Álvaro Regueira Fernández, José Ignacio Andrés Viloria, Diego Palenzuela Blasco, Gema Rodríguez Cerón, Ángel Fernández Collantes, Rocío López Herrero, Lucia Gil Gomez, Jesus Fernanz Anton, Veronica Gonzalez Santin, Eva Bassas Parga, Natalia Montero Gaig, Jordi Castellvi Valls, Laura Grau Torradeflot, Luisa Fernanda Cueva Castro, Jordi Genoves Gonzalez, Patricia Calvo Pasaron, Carlos Jerico, Khadidjettou Mohamed Yahadih, Ana Tejedor Navarro, Marta Garcia Martinez, Alex Romero Fernández, Sainz Lete, David Gomez Dominguez, Maria Jesus Sanz De Leon, Rodrigo Urruchi, Laura Vaquero, Patricia Rodriguez, Manuel Carazo, Alba Perez, Alvaro Gudina, Maria Jose Blanco, Irene Arranz, Delia Velasco, David Martin, Elena Laita, Maria Garcia, Marta Ramos, Esther Aguado, Eugenio Ruiz De Santos, Laura Leal, Alicia Bordell, Cristina Barbosa, Ester Mora, Antonio Navarro, Guillermo Puig, Inma Camprubi, Marta Caballero, Adriana Rueda, Miguel Victoriano Solis Sanchez, Maria Huerta Carrascosa, Juan Pablo Nocete Navarro, Elena Murcia Sanchez, Jose Javier Herrero Parras, Paula Rodriguez Nieto, Isabel De La Calle Gil, Maria Gonzalez Cofrade, Rosalía Navarro Casado, Miguel Cendrero Martin, Adrian Martinez Lopez, Gabriela Seronero Vasquez, Alvaro Ramiro Ruiz, Pablo Garcia Pimentel, Maria Labalde Martinez, Cristina Nevado Garcia, Javier Garcia Borda, Pablo Pelaez Torres, Alfredo Vivas Lopez, Oscar Garcia Villar, Tamara Llamero, Alicia Ferrer, Marta Gonzalez Bocanegra, Alba Manuel Vázquez, Ignacio Aguila Collantes Velasco, Myrna Assaf Ballut, Nuria Baeza Pintado, Paloma Sanz Munoz, Ana Garcia Tello, Juan Boronat Catala, Juliusz Jan Szczesniewski, Jose Ramon March, Marina Garcia Cogollo, Cristina Hernandez Avila, Ainhoa Valle Rubio, Jose Luis Ramos Rodriguez, Alberto Hernandez Matias, Jose Mariá Jover Navalon, Mercedes Martinez Garcia, Celddy Mireya Urquizo Torrico, Silvia Benito Barbero, Justyna Drewniak Jakubowska, David Stolle Duenas, Ana Nieto Moreno, Alfredo Abad Gurumeta, Paula Fernandez Valdes Bango, Alicia Ruiz Escobar, Diana Fernandez, Javier Ripolles Melchor, Elena Saez Ruiz, Elena Nieto Moreno, Norma Aracil Escoda, Elena Lucena De Pablo, Marina De Retes, Kateri Chao, Berenice Gutierrez Tonal, Eugenio Martinez Hurtado, Mercedes Garcia Dominguez, Barbara Algar Yanez, Paloma Munoz Saldana, Ana Maria Tirado Errazquin, Sandra Maria Gadin Lopez, Rut Salvachua, Beatriz Nozal, Cristina Sevilla Guillen, Rosa Sanz Gonzalez, Olga De La Varga Martinez, Marina Valenzuela Pena, Beatriz Vazquez Rivero, Patricia Serrano-Mendez, Alexander Forero-Torres, Fuad Lopez-Fernandez, Francisco J Reinoso, Clara Marchesi Alcober, Begona Ayas Montero, Sandra Verdeguer Ribes, Maria Jose Alberola Estelles, Ana Navarro Barles, Xenia Carre Rubio, Ildefons Sarda Aure, Xenia Carre Rubio, Marta Silvestre Cepeda, Enrique Ruiz Velasquez, Sami Galal Eldin, Wael Alkhaleel, Hala Alhomi Alsaid Mushaweh, Ahmad Alhaj, Marwan Al Aliwy, Marah Zeidani, Abdallah Aladna, Kusay Ayoub, Lama Kadoura, Mousa Sifat, Joudi Chachati, Ammar Niazi, Ehab Alaasy Alkushti, Hilal Matar, Abdalwahab Alkhalaf, Opadah Salhab, Masa Watfa, Alhasan Alkhayer, Habib Olaisheh, Ali Hammed, Ahmad Molhem, Ali Sulaiman Deeb, Naya Hassan, Bassam Hassan, Ali Esmael, Hamza Ibrahim, Zuhoor Almohanady, Ghadeer Al Wajeeh, Khadega Abotaleb, Waheeb Al Kubati, Ghofran Al Nahwi, Ashwaq Ziyad, Hanan Sheebren, Faris Alhajami, Hanadi Ali, Wejdan Al Wafi, Amani Altaweel, Belquis Aljailani, Salwa Al Eryani, Abeer Mohammed, Abeer Saadan, Bushra Alshaikh, Shada Alemad, Manal Aldhaheri, Qasim Aldhaheri, Rafat Al Saban, Suhail Alshehab, Heba Abduljawad, Maha Muharram, Amal Muharram, Eman Al Markiz, Sarah Shream, Hanan Alhakimi, Shaima Ziyed, Ansam Alrobaiee Al Thawra.

## Supplementary Material

znae158_Supplementary_Data

## Data Availability

Research data and other material (for example, study protocol, data analysis plan) will be available to the scientific community, immediately upon publication, with as few restrictions as possible. All requests should be submitted to the corresponding author for consideration.
