# Correction to: Postoperative anaemia increases unplanned readmission: an international prospective cohort study of patients undergoing major abdominal surgery

**DOI:** 10.1093/bjs/znaf118

**Published:** 2025-06-10

This is a correction to: Kenneth J Macpherson, POSTVenTT International Collaborative , Postoperative anaemia increases unplanned readmission: an international prospective cohort study of patients undergoing major abdominal surgery, *British Journal of Surgery*, Volume 111, Issue 7, July 2024, znae158, https://doi.org/10.1093/bjs/znae158

In the originally published version of this manuscript, the names of the collaborators Warren Seow and Laure Taher Mansour were inadvertently omitted from the collaborator list. This error has been corrected.

