# Supplementary material for: Postoperative anaemia increases unplanned readmission: an international prospective cohort study of patients undergoing major abdominal surgery
Source: Br J Surg. 2024 Jul 11;111(7):znae158. doi: 10.1093/bjs/znae158 (PMC11238848; doi:10.1093/bjs/znae158)
Supplement: znae158_Supplementary_Data [file znae158_supplementary_data.docx]

**Postoperative anaemia increases unplanned readmission: an international prospective cohort study of patients undergoing major abdominal surgery**

POSTVenTT International Collaborative*

**All collaborators are listed in the supplementary material below.*

**Corresponding author.**

Dr Kenneth J. Macpherson *MD*

[kenneth.macpherson@health.wa.gov.au](mailto:kenneth.Macpherson@health.wa.gov.au)

**ORCID ID**; 0000-0001-6822-6776

**Twitter** @kennethmacphe15

| **Supplementary Methods** |  |
| --- | --- |
| Patient Eligibility Criteria | *Page 2* |
| Procedure Eligibility Criteria | *Page 3* |
| **Supplementary Results** |  |
| Reasons for Readmission | *Page 6* |
| Preoperative Clinical Data  Patients by Country | *Page 7*  *Page 7* |
| **Supplementary Appendices** |  |
| Authorship list | *Page 8* |

**Supplementary Methods**

### Patient Eligibility **Summary**: The study population will include consecutive adult patients undergoing major emergency or elective abdominal surgery.

**Inclusion criteria:**

- **Age**: Adult, 18 years or above.
- **Procedure:** A major abdominal surgery is defined as an operation with an incision into the abdominal cavity and anticipated duration of more than one hour. Procedures performed using any surgical approach, including open, laparoscopic, and robotic surgery are included.
- **Urgency**: Patients undergoing planned (elective or expedited) or unplanned (emergency) surgery. **Exclusion criteria:**
  - **Procedures:** Abdominal surgery classified as minor operations such as; laparoscopic appendicectomy (emergency or elective), endoscopy procedures, transanal or transurethral procedures.
  - **Indication:** Palliative procedures as determined pre-operatively and explicitly stated in the medical record or consent form.
  - **Extent of surgery:** Operations that are either
    o staged with a planned return for re-operation (such as but not exclusively, damage control
    laparotomy or burns surgery).
    o Change in operative plan such that during the first procedure it is determined that a re-operation
    is necessary, even if the patient was enrolled pre-operatively
  - **Return to theatre**: Each patient should only be included in the study *once*. Patients returning to theatre due to complications following earlier surgery can be included, as long as their index procedure has not already been included in the POSTVenTT study.

| Included procedures: | |
| --- | --- |
| Specialty | Procedures |
| Transplant | Liver/Pancreas/Intestine/Kidney/Abdominal multi viscera |
| Upper GI | Oesophagectomy (any) |
|  | Total/Partial gastrectomy +/- excision of surrounding tissue |
|  | Other operations on small bowel including formation/reversal of ileostomy |
|  | Anti-reflux surgery |
|  | Hellers cardiomyotomy |
|  | Bariatric surgery |
| Colorectal | Caecectomy |
|  | Right hemicolectomy |
|  | Extended excision of right hemicolon/Extended right hemicolectomy |
|  | Excision of transverse colon |
|  | Excision of left hemicolon |
|  | Extended excision of left hemicolon/Extended left hemicolectomy |
|  | Excision fo sigmoid colon |
|  | Anterior resection |
|  | Hartmann’s procedure |
|  | Other operation on colon (bypass, colostomy) |
|  | Ileoanal anastomosis and creation of pouch |
|  | Total excision of colon and ileorectal anastomosis |
|  | Panproctocolectomy and ileostomy |
|  | Abdominoperineal resection |
|  | Abdominoperineal pull through resection with colo-anal anastomosis +/- colonic pouch and associated stoma |
|  | Reversal of Hartmann’s procedure / colostomy |
|  | Pelvic exenteration |
| Hepatic, pancreatic, biliary | Lap / Lap converted to open / Open cholecystectomy +/- exploration of CBD +/- cholangiogram |
|  | Pancreatectomy / Pancreatoduodenectomy (Whipple’s procedure) |
|  | Hepatojejunostomy |
|  | Liver resection |
|  | Splenectomy |
| Urology | Nephrectomy |
|  | Nephroureterectomy |
|  | Construction of ill conduit including ureteric implantation |
|  | Ill or colonic replacement of ureter |
|  | Partial / Total cystectomy + with construction of intestinal conduit or bladder |
|  | Enterocystoplasty / Enlargement of bladder / Bladder augmentation |
|  | Diverticulectomy of bladder |
|  | Repair of vesicocolic fistula |
|  | Radical prostatectomy, reconstruction of bladder neck including bilateral pelvic lymphadenectomy |
| Gynaecology | Radical hysterectomy and lymphadenectomy (Wertheim’s) |
|  | Hysterectomy with excision / biopsy and or removal of momentum and uterine adnexa for ovarian malignancy |
|  | Total / Subtotal abdominal hysterectomy (+/- oophorectomy) |
|  | Ovarian cystectomy +/- omental biopsy (as sole procedure and including bilateral) |
|  | Block dissection of pelvic lymph nodes (as sole procedure) |
|  | Myomectomy |
| Vascular | Open AAA repair |
|  | Aorto-bifemoral bypass |
|  | Neoaortic reconstruction |
| Abdominal Wall | Incisional hernia >1 |
| Misc | Multivisceral resections (defined as resections involving >/= 2 distinct parts of GIT or genitourinary or gynaecological (excluding ovaries only) or HPB) |

| Excluded procedures: | |
| --- | --- |
| Specialty | Procedures |
| Gastrointestinal | Gastroscopy / Colonoscopy / Sigmoidoscopy (diagnostic or therapeutic) |
|  | Diagnostic laparoscopy / Staging laparoscopy |
|  | Ascitic drain (drainage of peritoneal cavity) |
| Colorectal | Haemorrhoid operations |
|  | Anal fistula operations |
|  | Laparoscopic / Open appendicectomy |
| Hepatobiliary | Endoscopic biopsy / excision of HPB structures |
|  | Endoscopic retrograde cholangiopancreatography (ERCP) |
| Urology | Cystoscopy |
|  | Bladder biopsy |
|  | Percutaneous nephrostomy |
|  | Percutaneous nephrolithotomy (PCNL) |
|  | Total orchidectomy |
|  | Vasectomy |
|  | Insertion of ureteric stent |
|  | Transurethral resection of the prostate (TURP) |
|  | Transurethral resection of bladder tumour (TURBT) |
| Vascular | Transluminal (endovascular) procedures on arteries (diagnostic or therapeutic) |
| Abdominal Wall | Abdominal wall hernias including inguinal/inguinoscrotal/femoral (Lap or open including TEP or TAPP), only exception is incisional hernia > 1 hr |

**Supplementary Results**

| Supplementary Table 1: Reasons for Readmission | | |
| --- | --- | --- |
|  |  | Readmission |
| Total N (%) |  | 343 (6.8) |
| Days to Readmission | Median (IQR) | 10.0 (5.0 to 18.0) |
| Readmission Type | Planned | 28 (8.2) |
|  | Unplanned | 314 (91.8) |
| Complication during  Readmission | 1 | 33 (10.8) |
| (Clavien-Dindo Classification) | 2 | 107 (35.0) |
|  | 3 | 100 (32.7) |
|  | 4 | 61 (19.9) |
|  | 5 | 5 (1.6) |
| Reoperation during  Readmission | No | 285 (83.3) |
|  | Yes | 57 (16.7) |
| Reason for Readmission | Blood Transfusion or Iron Infusion | 5 (1.5) |
|  | Chemotherapy | 4 (1.2) |
|  | Endoscopy | 7 (2.1) |
|  | Infection - General | 57 (16.7) |
|  | Infection - Wound | 49 (14.4) |
|  | Other | 4 (1.2) |
|  | Other Procedure | 7 (2.1) |
|  | Post Operative Complication  - General | 156 (45.7) |
|  | Post Operative Complication  - Pain | 47 (13.8) |
|  | Removal of urinary catheter | 5 (1.5) |

| Supplementary Table 2 - Preoperative Clinical Data | | | | |
| --- | --- | --- | --- | --- |
|  |  | Readmission | No Readmission | p |
| Preoperative Clinical Frailty Score | Not Frail (1-3) | 247 (5.8) | 3988 (94.2) | <0.001 |
|  | Mild Frailty (4-5) | 77 (11.8) | 574 (88.2) |  |
|  | Moderate-Severe Frailty (6+) | 15 (11.6) | 114 (88.4) |  |
| Preoperative Anaemia | No Anaemia | 194 (5.9) | 3115 (94.1) | <0.001 |
|  | Anaemia | 129 (9.2) | 1267 (90.8) |  |
| Preoperative Haemaglobin | Mean (SD) | 126.7 (20.8) | 131.7 (18.7) | <0.001 |

| Supplementary Table 3 - Patients by Country | |
| --- | --- |
|  | Patients recruited (%) |
| Australia | 2044 (40.0) |
| Spain | 979 (19.2) |
| New Zealand | 685 (13.4) |
| Italy | 612 (12.0) |
| Egypt | 457 (6.9) |
| Libya | 113 (2.2) |
| Jordan | 85 (1.7) |
| Syria | 69 (1.3) |
| Yemen | 41 (0.8) |
| Palestine | 13 (0.2) |
| Sudan | 11 (0.2) |
| Oman | 3 (0.0) |
| Total | 5512 |

Supplementary Appendices

POSTVenTT Collaborators

### Writing Committee

Kenneth J. Macpherson, Ane Abad Motos, Vinicio Mosca, Ruth Blanco Colino, Muhammed Elhadi, Eman Ali, Cameron Wells, Chris Varghese, William Xu, Jurstine Daruwalla, Gianluca Pellino, Jose Antonio Garcia Erce, Nagendra N. Dudi-Venkata, Toby Richards

### Advisory Group

David I Watson, Pete Pockney, Deborah Wright, Toby Richards, Doug Robb, Guiliana D’Aulerio, Jani Lee Moss, Jess Vo, Jai Darvall, Matthew A. Warner, Nagendra N. Dudi-Venkata, Warren Seow, Laure Taher Mansour

### Trial Managers

Jayne Lim, Guiliana D’Aurelio

### Regional Leads

**Australia**: Su Kah Goh, David S Liu (Victoria); Muhammad Ibrahim (Queensland); Amanda C Dawson, Elizabeth WY Lun (New South Wales); Jurstine Daruwalla (Tasmania); Adam J Frankel (Queensland); Siang Wei Gan (South Australia); Jonathan Foo (Western Australia).

**Italy:** Francesco Pata, Gianluca Pellino

**Middle East & North Africa**: Muhammed Elhadi

**New Zealand**: Liam Ferguson

**Spain:** Ane Abad Motos*,* Gianluca Pellino

### Supervising consultants

**Australia**: Daniel Chubb (Albury-Wodonga Health); Christophe Berney (Bankstown); Sonalmeet Nagra (Barwon (Geelong)); Chonhann Liew, Hasanga Jayasekera (Bendigo); Merwe Hartslief (Cairns); Adeep Majid (Calvary Mater Newcastle); Henry Y Cheung (Concord); Sayed Hassen (Eastern Health); David I Watson (Flinders Medical Centre); Amanda C Dawson (Gosford Hospital); John VanBockxmeer (Hedland Health Campus); Tobias J Evans (Hobart Hospital); Francesco Amico (John Hunter); Girish Pande (Launceston Hospital); Justin S Gundara (Logan Hospital); Elizabeth Murphy, Bernd Froessler (Lyell McEwin Hospital); Sarah Martin (Monash (Casey)); Daniel Croagh (Monash (Clayton)); William Teoh (Monash (Dandenong)); Matthias Wichmann (Mount Gambier and Districts Health Service); Jonathan Fong (North West Regional Hospital); Krinalkumar Mori (Northern Health); Quentin Ralph, Aliakbar Estakhri (Port Lincoln Health and Hospital Service); Adam J Frankel (Princess Alexandra Hospital); Sarah R Bowman (QE II (Jubilee)); Harsh Kanhere (Royal Adelaide Hospital); Benjamin Loveday (Royal Melbourne); Stephanie Chetrit (Royal Perth Hospital); Dale Currigan, Jonathan Foo (Sir Charles Gairdner Hospital); Mary Theophilus (SJOG Midland); Michael J Johnston (St Vincent); Markus Trochsler (The Queen Elizabeth Hospital); Justin Yeung (Western Health (Footscray)); Guy Maddern (Whyalla Hospital & Health Services and Port Augusta); Richard G McGee (Wyong Hospital)

**Egypt:** *Mohamed Asal (Alexandria Main University Hospital);* *Mostafa Abbas (Assiut University Hospital);* *Marcello*

***Italy****: Giuseppe Spampinato (“Vito Fazzi" Hospital);* *Donato F Altomare (AOUC Policlinico di Bari);* *Piergiorgio Danelli (Azienda Ospedaliera Universitaria L. Sacco, Milano);* *Andrea Mingoli (Azienda Ospedaliera Universitaria Policlinico Umberto I, Roma);* *Simone Giacopuzzi*, *Luca A. Fumagalli (Ospedale A. Manzoni, ASST Lecco);* *Stefano Olmi (Policlinico San Marco GSD, Zingonia);* *Marzia Franceschilli (Policlinico Tor Vergata Hospital);* *Adolfo Pisanu (Policlinico Universitario di Monserrato 'Duilio Casula’);*

**Jordan:** *Ghaith Bani Abdel Rahman (Al-Basheer Hospital);*

**Libya:** *Milad Gahwagi (Benghazi Medical Center)*

***New Zealand****: Ian Bissett (Auckland); Jeremy Rossaak (Bay of Plenty); Saxon Connor (Canterbury); Alexander W Brown (Capital and Coast); Andrew MacCormick, Sze-Lin Peng (Counties Manukau); Peter Stiven (Gisborne Hospital); Bevan Jenkins (Hawkes Bay); James Tietjens (Hutt Valley); Christopher Harmston (Northland); Christopher M Gray (South Canterbury); Deborah M Wright, James G Wilkins (Southern DHB (NZ)); Jasen Ly (Waikato); Andrew Herd (Waitemata); Sarah Rennie (Wangaratta Hospital); Marianne Lill (Whanganui).*

**Palestine:** *Raed Amro (Abu AL-Hasan AL-Qasim hospital)*

**Spain:** *Jose Ramon Rodriguez Fraile (Guadalajara University Hospital);* *Maria Moral Gonzalez (Hospital Universitario de Mostoles);* *Santiago Valderrabano-Gonzalez (Hospital Universitario La Paz)*

***Sudan:*** *Montasir A Abdalbain (Al-Moalem Medical City)*

***Syria:*** *Ahmad Ghazal (Aleppo University Hospital);*

**Yemen:** *Nashwan Tashan, Mohammed Al Shehari (Al Thawra Modern General Hospital);*

### Local Leads

***Australia:*** *Jed Hughes (Albury-Wodonga Health); Joseph C Kong (Alfred); Melanie E Cusso (Armadale Hospital); Daniel R Cox (Austin Health); Sarit S Badiani (Bankstown); Sara Mohammed Jinnaah (Barwon (Geelong)); Amy Crowe (Bendigo); Muhammad Ibrahim (Cairns); Henry Y Cheung (Concord); Enoch Wong (Eastern Health); Uyen G Vo (Fiona Stanley Hospital); Melissa Y Wee (Flinders Medical Centre); Amanda C Dawson, Elizabeth WY Lun (Gosford Hospital); Matthew J Marino (Hobart Hospital); Isabella Ludbrook; Anne- Marie Aubin (John Hunter); Jurstine Daruwalla (Launceston Hospital); Justin S Gundara (Logan Hospital); Timothy J Ganguly (Lyell McEwin Hospital); Tara Kannan (Maitland Hospital); Fiona Pavan (Monash (Casey)); Geraldine J Ooi (Monash (Clayton)); Vignesh Narasimhan (Monash (Dandenong)); Paul T Heitmann (Mount Gambier and Districts Health Service); Tiffany J Cherry (North West Regional Hospital); Sharon Lee (Northern Health); Teng-Wei Khoo (Port Augusta Hospital and Regional Health Services); Adam J Frankel (Princess Alexandra Hospital); William Lindores, Sarah R Bowman (QE II (Jubilee)); Siang Wei Gan (Royal Adelaide Hospital); Christine Li (Royal Melbourne); Xiao-Ming S Woon-Shoo-Tong (Royal Perth Hospital); Amelia L Davis (Sir Charles Gairdner Hospital); WeiShearn Poh (SJOG Midland); Thomas Tiang, Victor W Chai (St Vincent); Gayatri Asokan (The Queen Elizabeth Hospital); Eunice Tse (Western Health (Footscray)); Brianne Lauritz (Western Health (Sunshine)); Alicia Lim (Whyalla Hospital & Health Services); Richard G McGee (Wyong Hospital)*

***Egypt:*** *Mahmoud Shaban Abdeljalil (Ain Shams University hospitals (El-Demerdash Hospital);* *Moataz Ewedah (Alexandria Main University Hospital);* *Ahmed Abbas, Marian Abou El Garad (Assiut University Hospital);* *Hossam Elfeki (Mansoura University Hospitals);* *Mohamed Elbahnasawy (Tanta University Hospital);*

**Finland:** *Joonas H Kauppila (Oulu University Hospital)*

***Italy:*** *Stefano Garritano (“Vito Fazzi" Hospital);* *Arcangelo Picciariello (AOUC Policlinico di Bari);* *Francesco Colombo (Azienda Ospedaliera Universitaria L. Sacco, Milano);* *Pierfrancesco Lapolla (Azienda Ospedaliera Universitaria Policlinico Umberto I, Roma);* *Paola De Nardi (IRCCS Istituto scientifico San Raffaele, Milan);* *Mauro Zago (Ospedale A. Manzoni, ASST Lecco);* *Matteo Uccelli (Policlinico San Marco GSD, Zingonia);* *Giuseppe Sica* (Policlinico Tor Vergata Hospital); *Mauro Podda (Policlinico Universitario di Monserrato 'Duilio Casula’)*

***Jordan:*** *Almu’atasim Khamees (Al-Basheer Hospital)*

***Libya:*** *Milad Gahwagi, Wafa Aldressi (Benghazi Medical Center);* *Eman Abdulwahed* (Tripoli Central Hospital)

***New Zealand:*** *Jelle M Vander Have (Auckland); Imogen I Watt (Bay of Plenty); Brodie M Elliott (Canterbury); Chelsea A Allen-Brough (Capital and Coast); Carys Finlayson (Counties Manukau); Olga Korduke (Gisborne Hospital); Mathew Morreau (Hawkes Bay); George N Weeratunga (Hutt Valley); Matthew J McGuinness (Northland); Jethro Palmer (South Canterbury); David C Kieser (Southern Cross Hospital); Xavier Field (Waikato); Dhenisha Dahya (Wairarapa); William Xu (Waitemata); Louis-Antoine Bonnet (Whanganui).*

**Oman:** *John George Grace Massoud (Khoula Hospital)*

***Palestine:*** *Sarah Amro (****Abu AL-Hasan AL-Qasim hospital****)*

**Spain:** *Fátima Cañeque Yanini (ES, Principe Asturias Hospital); Beatriz Amoros* (Guadalajara University Hospital) *Aranzazu Calero Lillo (Holy Spirit Hospital Foundation); Misericordia Basora (Hospital Clínic de Barcelona);* *Rosalia Navarro Perez (Hospital Clínico San Carlos);* *Rita Pilar Rodriguez Jimenez (Hospital Clínico Universitario Valladolid);* *Carmen Deiros Garcia (Hospital de Sant Joan Despí Moisès Broggi);* *Aitor Landaluce Olavarria (Hospital de Urduliz);* *Cesar Aldecoa (Hospital Rio Hortega);* *Montse Mallol Oliva (Hospital Universitari de Bellvitge);* *Raquel Garcia Alvarez (Hospital Universitario 12 de Octubre);* *Virginia Jiménez Carneros (Hospital Universitario de Getafe);* *Raquel Fernandez Garcia (Hospital Universitario de Mostoles);* *Ane Abad Motos (Hospital Universitario Infanta Leonor);* *Hanna Perez-Chrzanowska (Hospital Universitario La Paz);* *Maria Vila Montanes (Hospital Universitario y Politécnico La Fe);* *Maria Luisa Pinana Campon (Sant Joan de Reus University Hospital);*

**Sudan:** *Hytham K S Hamid (Al-Moalem Medical City)*

***Syria:*** *Ruqaya Masri (Aleppo University Hospital)* *Abdulrahman Almjersah*, *Ali Alloush (Tishreen University Hospital)*

**Yemen:** *Fatima Al Eryani (Al Thawra Modern General Hospital)*

### Data collectors

***Australia:*** *Jonathon Holt, Stephanie Potenza, Avelyn Wong, Sagarika Handa, Virginia Su, Aniqa Hussain, Angus Tolhurst-McKiernan, Amelia J Cooper, Mark T Butorac, Hugh Elbourne, Emily Dutta-Powell, Neave Kapoor, Caitlin Reid (****Albury-Wodonga Health****); Jason Wang, Stella Le, Jordi Elliott, Bill Wang, Jiting Li, Zhuoning Song, Aya Basam, Branavan Thevashangar, Shani Nguyen, Arsheeya Rattan, Jeffrey Shao, Cynthia Wells, Emma Aldous, Siobhan Brodrick, Brooke Callahan, Faizah Alam, Laura Casey, Wilson Yang, Swetha Prabhakaran, Haini Jiang, Alex Lin, Angela Chau, Pranjal Chaurasia, Morgan Jones, Yit J Leang, Wendy Brown, Jonathan Serpell, Peter Carne, Matthew Stokes (****Alfred****); Jern Cabral, Miriam W Bennamoun, Suneha Lal, Kai Dong Choong (****Armadale Hospital****); Yanran Yu, Chris Zhao, Vijayaragavan Muralidharan, Sean Stevens, Liam Fernando-Canavan, QiRui Soh, Nakjun Sung, Grace Gold, Alex Grogan, James G Lin, Ahmad Aly, Georgina Riddiough, Hamza Ashraf, Michaela Wojno, Adrian Yeoh, Gabriel Wall, Jack Liu, Sonia Gill, Yida Zhou, Robert Torode, Michael Issa, Jasun Li, Ruben Schuringa, Marcos V Perini, David Proud (****Austin Health****); Kevin Jia, Suhasini Shanmugarajan, Gladys Tam, Ashley Wang (****Bankstown****); Shipra Sankpal, Matthew Heard, Yung-Hsin Hsueh, Scott Anderson, Vishwakar Panuganti, Zoe Zhang, Gabriel Lirios, Joshua Birnie, Benjamin Scott, Lei Ying Barwon (****Geelong****)); Laura LeHua He, Samantha Mitchell, Chloe Wu, Brett Larner, Jason Fu, Amy McCormick, Daryn Goodwin (****Bendigo****); Alistair Lau, William Stockley, Ripudaman Jalota, Hannah Gora (****Cairns****); Parvathy Ananthan (****Calvary Mater Newcastle****); Thomas Estephan, Aakash Nanda, Sarika Suresh, Thihoai Trang Nguyen, Sarah McLain, Sharanya D Mohan, Nicole Batten, Gajanth Sivapalan, Grace S Ting, David Yoon, Joohyen Ahn, Lisa Shi (****Concord****); Salena Ward, Felicity Huang, Boya MacKeddie, Adrian Fox, JiYue Wang, Amie Hilder, Ashan Kathriachchige, Chen Lew, King Tung Cheung, Sarah Goh, Balakavitha Balaravi, Simran Vinod Benyani, Chris Ip, Shomik Sengupta, Christopher Steen, Janindu Goonawardena, Shantanu Joglekar, Erica Wang, Olivia Lin, Michelle Yao, Vinna An, Connor Burley, Raaj Chandra, Anshini Jain (****Eastern Health****); Amelia Davies, Matthew Mann, Jana-Lee Moss, Kenneth J Macpherson, Anand Trivedi, Matthew J Pugliese, Ron Ramos, Davina Daudu, Tasvinder Hans, Audrey Kim, Aiden Stuart, Harvinder Hans, Natalie Layden, Pravin Viswambaram, Ashley Lee, Mohammed Al-Zubaidi, Dickon Hayne, Hayley J McMillan, Jason D’Silva (****Fiona Stanley Hospital****); Ben P Finlay, Rebecca Fong, Penelope Peres, Gavin Nair, Suchitra KrishnanPillai, Yong Zhi Beh, Steven L Due, Benjamin Howes, Dominic Parker, Christina Mcveay, Matthew Marshall-Webb, Izhar-Ul Haque, AbdulAhad Rana (****Flinders Medical Centre****); Sneha E Ninan, Gemma J Qian, Joseph W Fletcher, Brendan Watkins, Luke C Green, Yara A Elgindy, Elodie V Honore, Charles J Roth, Jonathan Tandjung, Adam R Perkovic, Luca Borruso, Victor Yu, Thomas-Hoang Le, Samuel M Mathias, Daniel Jeong, Charlotte E Cornwell, Emily A Taylor, Kie Ren Oon (****Gosford Hospital****); Mikayla Everuss, Jesse Hafner, Caleb Kim (****Hedland Health Campus****); Catherine T Cartwright, Aram Cox (****Hobart Hospital****); Ricci Amolis, Hui Kang Tan, Brittany Harrop, Theepiga Ramachandran, Elli Farrell, Saxon Farquhar, Felicity Goodes, Sophie Zhong, Sophie Kelly, Howard Li, Jun Jie Chau, Joshua Richards (****John Hunter****); Christopher Cheong, Tedman Chau, James Pankhurst, Darren J Rama, Alicia Liong (****Launceston Hospital****); Sebastian Primrose, Peita M Webb (Logan Hospital); Edward Young, Harleen Kaur, Xuanyu Zhou, Mohamed Afzal, Jess Barnes, Allan Butler, Priya Nandoskar, Matt Besley, Mozafer Rajabali, Nikhil Kundu, Alexander Buckby, Linda Sunny, Olivia Anderson, Hemachalam Subramaniam (****Lyell McEwin Hospital****); Sandeep Dayaratne, Anjelee Segaran, Daniel Chilton (****Maitland Hospital****); Chris J Kim, Dion Koh, Tom A Eldredge, Syeda T Saief, Kevin K Yoong (****Monash (Casey)****); Ashray Rajagopalan, Kathleen Wisbey, Chelsea Lin, Jolene Ong, Sandra Li, Jonathan Tiong, Naman Kohli, Joseph Jaya, Jiakai Yao, Evie Yeap (Monash (****Clayton****)); Katherine Grant, Genevieve Heath-Kalgutkar, Ryan R Gosavi, David Zula, Ning Yih Kam, Travis Ackermann, Darryl Lee, Sean Lim, Alice Lee, Phi Nguyen, Lily Bae (****Monash (Dandenong)****); Roshini Nadaraja (****North West Regional Hospital****); David Duong, Emma Payne, Natalie Nguyen, Gabrielle Cutler (Northern Health); Fellicia Stanzah (****Port Augusta Hospital and Regional Health Services****); Matthew Watson, Luke Green (****Port Lincoln Health and Hospital Service****); Aveechal Prasad, Laxmi Sistla, Emily Overton (****Princess Alexandra Hospital****); Monika A Wojt, Amos Moody, William Zore, Rebecca Blundell (****QE II (Jubilee)****); Tarik Sammour, Syeda Khadijah Ahmed, Yickho Lam, EuLing Neo, Anthony Farfus, Leigh Warren, Zachary Bunjo, Jaspreet K Sandhu, Qiwen Wang, Syeda Khadijah Ahmed, Nimantha Abey, Victoria Lu, Amanda Hii, Shalvin Prasad, Christopher Dobbins, Mohammad Mangi, Joseph Dawson, Danielle Boniface, Mathew Amprayil (****Royal Adelaide Hospital****); Patwinder Gill, Jonathan McCafferty, S.f.michelle Ku, Zijiang Yang, Ming Z How, Frank Du, Reece Ansaar, Chelsea Xu, Nyan S Lwin (****Royal Melbourne****); Supisara Suk-Udom, Nathan Scott, Kyle Raubenheimer, Nivedan Jeyamanoharan, Priyanka Chakraborty, Bryce Stewart, Dinethma Tharuni Ekanayake, Diana Xiao, Aiswarya Ajith, Mark Ibraheem, Joseph Hanna, James Dodd, Sophie Zhao, Aine O’Dwyer, Sinali Seneviratne, Lauren Masi, Hilary Leeson, Xiao-Ming S Woon-Shoo-Tong (****Royal Perth Hospital****); Ho-Cing Yau, Daniel Lee, Siavash Mortazavi, Stephanie Bartley, Zheng DaChok, Nur Sabrina Binti Babe Azaman, Leesha Bryan, Bethany Furfaro, Peng Sheng Tan, Talia Shepherd, Mary Teoh, Kelly Shepherd (****Sir Charles Gairdner Hospital****); Danielle Malatzky , Nicole Hew, Pia J Schelfhout, Rama Chidambaram, Ashwita S Vanga, Madiha K Bajwa, Aidan D Yuen-Oye, Darren Karadimos, Nyssa Jualim, Vivien Hsu, Sharie A Apikotoa, Cathy Maher, Emalee Burrows (****SJOG Midland****); Natalie Yu, Michael W Hii, Lillian Taylor, Lynn Chong, Louis J Sisk, Cathy Hua, Elaine Su, Aidan Jackson, Elizabeth Hu, Tori Wong, Andrew Xu, Toni Zhang, Luke Bradshaw (St Vincent); Kathleen Nagle, Antonio Barbaro, Lata Limbu, Mai Nguyen, Kalyanasundaram (****The Queen Elizabeth Hospital****); Colin Choi, Ha M Nguyen, Shyamolie Mathur, Nicholas Quirk, Carol Z Wu, Reshi Suthakaran (****Western Health (Footscray)****); Chui Foong Ong, Ali Baker (****Western Health (Sunshine)****); Rosalina Lin, Jade T Bahnisch, Haili Luo (****Wyong Hospital****).*

***Egypt:*** *Mohamed Qassem*, *Omar Saeed Anas*, *Omar Abdelnasser Mohamed*, *Basil Alshami*, *Mariam Kefah Fathi*, *Muhammed Ahmed Muhammed Abdelrazeq*, *Hajar Fawzy Abdel Rady*, *Mahmoud El Garhy*, *Laila Raafat Mabrouk*, *Balqees Alhariri*, *Ahmed K Awad*, *Merihan A Elbadawy*, *Mahmoud Elsayed Abdelazeem Bakheet*, *Nadien Mohamed Abd El Hamid*, *Noha Eslam Attia Sakr*, *Yara Mohamed Yasser*, *Mostafa Osama Farag*, *Selwan Mahmoud Abdelrahman*, *Israa Mohamed Iraqui*, *Dalia Shaban Abdelgalil*, *Sherein Diab*, *Peter Nader Sobhi Rafla*, *Steven Danial Azmy Habib*, *Aya Abdulilah Abdulmonem*, *Bassant Hassan Shawki*, *Salma Adel Mohamed*, *Sally Ahmed Abd El Razik*, *Ahmed Hassan A rady*, *Ayatallah Ali Hussein Ramadan*, *Ayman K Awad*, *Pavly Wagih Beshay*, *F r s Gad*, *Salma Tarek Helmy*, *Michael Amin*, *Mahmoud Mohamed Mohamed Shalaby*, *Abanoub Ashraf Moufid Riyad* ***(Ain Shams University hospitals (El-Demerdash Hospital)****);* *Ahmed Nafea*, *Marina Hany*, *Ahmed Sakr*, *Mohamed Al Sayed*, *Mosaab Tayiawi*, *Mohammed Eleleigy*, *Osama Elzankaly*, *Mohamed Mourad*, *Shaher Shokralla*, *Yomna Dean*, *Yousef Tanas*, *Samar Abdelnaby*, *Abdulrhman Abdulhameed* ***(Alexandria Main University Hospital);*** *Afnan Morad*, *Esraa Emad Abdelazeem*, *Manal Abdelghany*, *Yosra Rateb*, *Eithar Alqady*, *Shereen Elnokrashy*, *Mona Helbawi*, *Asmaa Shaltout*, *Ahmed Hussein*, *Amira Salem*, *Ramy Hassan*, *Magdy Mahdy*, *Marina Adly*, *Sarah Abdelmoneim*, *Ahmed Taha*, *Ashraf Elnaggar*, *Mohamed Elkady*, *Omar Soliman*, *Ahmed Aboelmagd*, *Mahmoud Shehata*, *Shymaa Abodahab*, *Rehab Mohamed*, *Ahmed Youssef*, *Radwan Torky*, *Mohamed Abulfetouh*, *Gihan Galal*, *Sara Kalaifa*, *Mahmoud Khalil*, *Islam Ibrahim*, *Ahmed Kamel Morsi*, *Ahmed Wahba*, *Islam Abo Deef*, *Ahmed Abd Elzaher*, *Alaa Sultan*, *Abdallah Rashad Temerik*, *Ahmed Abdelmoneim*, *Shereen Saad*, *Shaymaa Ibrahim*, *Ahmed Elkoussy*, *Yasmine Adel Mohammed*, *Roaa Saleh*, *Randa El Zohne*, *Mohamed Shaker*, *Fatma Gharib*, *Esraa Kotb*, *Eman Hassan*, *Islam Aziz (****Assiut University Hospital);*** *Mohamed A Abdelmaksoud*, *Ahmed Ramadan*, *Reem Elsaadany*, *Mohamed Taman*, *Karim Elkenawi*, *Esraa Daif*, *Hossam Elsonny*, *Rahma Elboraei*, *Mahmoud Laymon*, *Osama Ezzat*, *Ahmed Elbatta*, *Mohamed Hassan*, *Amr A Elsawy*, *Ahmed Elsheshtawy*, *Islam Mansour*, *Amgad Fouad*, *Amr Sanad*, *Amr Kassem*, *Ahmed Beah*, *Ahmed Shehta*, *Ahmed E Nabih*, *Rana Nagi*, *Mostafa Shalaby*, *Ahmed Sakr*, *Yousef Hesham*, *Sameh Emile*, *Aly Sanad*, *Ahmed Elsayed*, *Mirna Sadek (****Mansoura University Hospitals);*** *Omar Taha*, *Shady Elsalhawy*, *Mustafa Zaghloul* ***(Tanta University Hospital)****;*

***Finland:*** *Olli Helminen*, *Heikki Huhta*, *Matti Aleksi Mosorin*, *Henri Juhani Sova*, *Ville Virta (****Oulu University Hospital)***

***Italy:*** *Norma Depalma*, *Gabriella Lionetto*, *Stefano D ugo*, *Tiziana Marchese*, *Farshad Manoochehri*, *Federico Perrone* ***(“Vito Fazzi" Hospital);*** *Rigers Dibra*, *Giuseppe Trigiante*, *Gennaro Martines*, *Luciano Rella*, *Marco Vulpi*, *Pasquale Ditonno*, *Giuseppe (****Lucarelli AOUC Policlinico di Bari);*** *Francesco Cammarata*, *Luca Ferrario*, *Andrea Bondurri*, *Alessandro Michele Bonomi*, *Anna Maffioli*, *Isabella Pezzoli*, *Albert Troci*, *Franco Palmisano*, *Andrea Gregori*, *Giacomo Piero Incarbone (****Azienda Ospedaliera Universitaria L. Sacco, Milano);*** *Giancarlo D ambrosio*, *Francesco Pata*, *Andrea Picchetto*, *Elena De Stefani*, *Gioia Brachini*, *Flavia Ciccarone*, *Ilaria Clementi*, *Giuseppe Marenga*, *Piero Bartolucci*, *Gennaro Mazzarella*, *Bruno Cirillo*, *Immacolata Iannone*, *Cristina De Padua*, *Enrico Fiori*, *Paolo Sapeinza*, *Simona Meneghini*, *Martina Zambon*, *Giulia Duranti*, *Costantino Leonardo*, *Giorgio Franco*, *Pierfranco Maria Cicerchia*, *Luigi Simonelli*, *Giovanni Battista Fonsi*, *Sara Giovampietro*, *Edoardo De Meis*, *Monica Mansi*, *Giovanni Casella*, *Lidia Castagneto Gissey*, *Anna Falasca*, *Catia Gugliuccello*, *Barbara Binda*, *Maura Rocchetti*, *Sara Santillo*, *Federica Scarno* (**Azienda Ospedaliera Universitaria Policlinico Umberto I, Roma);** *Andrea Vignali*, *Simonetta Massaron (****IRCCS Istituto scientifico San Raffaele, Milan);*** *Marco Ee Chiarelli*, *Fulvio Tagliabue*, *Alessia Malagnino*, *Giovanni Pesenti*, *Carmine Sciorio*, *Alessandro Maletta*, *Michele Morelli*, *Antonio Pellegrino*, *Claudio Reato*, *Giani Sofia (****Ospedale A. Manzoni, ASST Lecco);*** *Giusi Giulia La Manna*, *Andrea Martina Guida*, *Bruno Sensi*, *Vittoria Bellato*, *Giuseppe Tisone*, *Roberta Angelico*, *Tommaso Maria Manzia*, *Leandro Siragusa*, *Giuseppe Farullo*, *Luca Orecchia*, *Lorenzo Alteri*, *Virgilio Michael Ambrosi Grappelli (****Policlinico Tor Vergata Hospital);*** *Tiziana Pilia*, *Valentina Murzi*, *Gianmarco D ancona*, *Stefano Angioni*, *Monica Ortenzi*, *Maurizio Nicola D alterio*, *Alessandro Cois*, *Marcello Pisano (****Policlinico Universitario di Monserrato 'Duilio Casula’);***

***Jordan:*** *Luai Abu Ismail*, *Younis Maher*, *Abdallah Yasin*, *Aseel Almohtasib*, *Farah Al Kasaji*, *Saja Aloran*, *Lana Sbitan*, *Heba Abu Khalaf*, *Subhi Al Issawi*, *Radi Tofaha Alhusseini*, *Sajeda Awadi (****Al-Basheer Hospital)***

***Libya:*** *Issa A. Abuzeid*, *Sarah Aldressi*, *Mohammed Alabeedi*, *Mohammed Moftah*, *Eman Salem*, *Mohammed N Albaraesi*, *Haitham Elmehdawi*, *Ahmed Ahmayda*, *Ala Burgeia*, *Tarik Eldarat*, *Maruwan Safi Abdullah*, *Abraheem Aljibali*, *Rema Benhariz*, *Almotasem Bellah Elsharif*, *Yasmin Abu Ihlayqah*, *Intesar Elmejrisi*, *Fatimah Abouthiheer*, *Rabha Ibrahim*, *Ayoub N Abdalmaged Akwaisah*, *Suha Younis Hasan*, *Ehsan Al Mahdi (****Benghazi Medical Center);*** *Ehtuish Farag Ehtuish*, *Mohamed Alsori*, *Sultan Ahmeed*, *Sanad Kanna*, *Entisar Alshareea*, *Enas Soula*, *Sarah Aljamal*, *Ehda Kerwash*, *Doaa Gidiem*, *Ekram Abujrad*, *Hayat Ben Hasan*, *Aamal Askar*, *Esmail Hamuda*, *Khawla Derwish*, *Reem Ghmagh*, *Abdulqudus Deeknah*, *Fras Elhajdawe*, *Marwa Sinan (****Tripoli Central Hospital)***

***New Zealand:*** *Jim Hsu-Shun Wang, Binura Lekamalage, Arpita Bansal, Luke DeRuiter, Kevin Yi, Faseeh Zaidi, Cindy Xu, Cain Anderson, Gabrielle Murti (****Auckland****); Natasha P Mitchell, Celine Peng, Olivia Mackay, Daniel A Carson, Sameer Bhat, Jenny Choi (****Bay of Plenty****); Justin Roake, Oliver Lyons, Eric T Lim, MeiSze Lee, Jared McLauchlan, Claire Whitehead, Sandra J Campbell, Fiona Carey, Grace Gatenby, Angharad Jones, Tamara Glyn, Heidi Blackburne (****Canterbury****); Emilie Roberts, Lauren Siggins (****Capital and Coast****); Chris Varghese, Renato Pitesa, Braedon Epps, Emma Espiner, Jack Hanlon, Ben Waller (****Counties Manukau****); Abhinav Bhasin, Sharmaine Sreedhar, Lauren Hubbard, Emily Evison (****Gisborne Hospital****); Molly Reynolds, Harriet Marshall, Sam Guest, Hinerangi Temara, Alexa Kuffel (****Hawkes Bay****); Wing Yung Agnes Chu, Rebecca Teague (****Hutt Valley****); Rowan Auchterlonie, Vilingatoni Hasiata, Benjamin Keren, Joshua Tiro (Northland); Gabriella Giacon (****South Canterbury****); Kushan Gandhi, Jeffrey Huang, Omar Mohyieldin, Alexander Hart, Mostafa Amer (****Southern DHB (NZ)****); Kate Dunstall, Tina Zhang, Ria George, Sang Ho Kim (****Waikato****); Stevie Waerea, Kate Henley (****Wairarapa****); Daniel Wen, Haru Yasutomi, Isaac Samuels, Ana Menon, Boaz Competente (****Waitemata****); Michael O’Grady, Thitapon Uiyapat (****Whanganui****).*

**Oman:** *Mazoon Said Ahmed Al Jabri (****Khoula Hospital)***

**Spain:** *Luis Bejarano Redondo*, *Cristina Lasa Unzue*, *Raquel Chaves Lopez*, *Beatriz Ledesma Muñoz*, *Irene Santos Prado*, *Maria Angeles Mancebo Zafra*, *Laura Hughes Lopez*, *Sahely Flores Risco*, *Daniel Marivela Palacios*, *Alvaro Martos Polo*, *Laura Soriano Melero*, *Hugo Eduardo Martin Acero Perez*, *Javier Feito Sancho*, *Raquel Torrillas Lopez*, *Guillermo Ocaña Ojea*, *Ana Isabel Vidal Conde*, *Irene Sastre Yague*, *Alvaro Revuelta Rebollo*, *Magali Ruiz Medrano*, *Elena Fayos Herrera (****ES, Principe Asturias Hospital****);* *Raquel Munoz*, *Carrmen Ramon*, *Mercedes Cabellos*, *Coral Ramirez (****Guadalajara University Hospital);*** *Enrique Lopez Ruiz (****Holy Spirit Hospital Foundation);*** *Marta Ubre*, *Julia Martinez Ocon*, *Guillermo Laguna*, *Raquel Risco*, *Purificacion Matute*, *Manuel López-Baamonde*, *Marina Vendrell*, *Pinar De Santos*, *Beatriz Tena*, *Paula Masgoret*, *Guido Munoz* ***(Hospital Clínic de Barcelona);*** *Juan Vidal Diaz*, *Lourdes Duran*, *Adriana Monente*, *Angela M De Molano*, *Brezo Del Blanco*, *Irene Murguialday*, *Juanjo Sanchez Palomo*, *Estrella Valencia*, *Blanca Carrillo*, *Marta Embid*, *Victoria Acedo*, *Nelia Tabatabaian*, *Ruben Sanchez*, *Pelayo Cadenas*, *Vlada Bizga*, *Alejandra Lasprilla*, *Carlos Aguilar*, *Rosa Beltrao*, *Tania Fernandez*, *Ana Mcg*, *Pedro Calle*, *Maria Jose Reyes*, *Kevin Romero*, *Guillermo Martin*, *Nacho Nunez*, *Luisa Bolivar*, *Jaime Rodriguez (****Hospital Clínico San Carlos)****; Rocío Gutiérrez Bustillo*, *Julia Martín-Romo Capilla*, *Juan Manuel Priede Vimbela*, *María José Cobreces García*, *Álvaro Regueira Fernández*, *José Ignacio Andrés Viloria*, *Diego Palenzuela Blasco*, *Gema Rodríguez Cerón*, *Ángel Fernández Collantes*, *Rocío López Herrero (****Hospital Clínico Universitario Valladolid);*** *Lucia Gil Gomez*, *Jesus Fernanz Anton*, *Veronica Gonzalez Santin*, *Eva Bassas Parga*, *Natalia Montero Gaig*, *Jordi Castellvi Valls*, *Laura Grau Torradeflot*, *Luisa Fernanda Cueva Castro*, *Jordi Genoves Gonzalez*, *Patricia Calvo Pasaron*, *Carlos Jerico*, *Khadidjettou Mohamed Yahadih*, *Ana Tejedor Navarro*, *Marta Garcia Martinez*, *Alex Romero Fernández (****Hospital de Sant Joan Despí Moisès Broggi);*** *Begoña Estraviz*, *Mario De Francisco Rios*, *Ane Emaldi Abasolo*, *Aitor Sainz Lete*, *David Gomez Dominguez* (**Hospital de Urduliz**); *Maria Jesus Sanz De Leon*, *Rodrigo Urruchi*, *Laura Vaquero*, *Patricia Rodriguez*, *Manuel Carazo*, *Alba Perez*, *Alvaro Gudina*, *Maria Jose Blanco*, *Irene Arranz*, *Delia Velasco*, *David Martin*, *Elena Laita*, *Maria Garcia*, *Marta Ramos*, *Esther Aguado*, *Eugenio Ruiz De Santos*, *Laura Leal*, *Alicia Bordell*, *Cristina Barbosa* (**Hospital Rio Hortega**); *Ester Mora*, *Antonio Navarro*, *Guillermo Puig*, *Inma Camprubi*, *Marta Caballero*, *Adriana Rueda* (**Hospital Universitari de Bellvitge**); *Miguel Victoriano Solis Sanchez*, *Maria Huerta Carrascosa*, *Juan Pablo Nocete Navarro*, *Elena Murcia Sanchez*, *Jose Javier Herrero Parras*, *Paula Rodriguez Nieto*, *Isabel De La Calle Gil*, *Maria Gonzalez Cofrade*, *Rosalía Navarro Casado*, *Miguel Cendrero Martin*, *Adrian Martinez Lopez*, *Gabriela Seronero Vasquez*, *Alvaro Ramiro Ruiz*, *Pablo Garcia Pimentel*, *Maria Labalde Martinez*, *Cristina Nevado Garcia*, *Javier Garcia Borda*, *Pablo Pelaez Torres*, *Alfredo Vivas Lopez*, *Oscar Garcia Villar* (**Hospital Universitario 12 de Octubre**); *Tamara Llamero*, *Alicia Ferrer*, *Marta Gonzalez Bocanegra*, *Alba Manuel Vázquez*, *Ignacio Aguila Collantes Velasco*, *Myrna Assaf Ballut*, *Nuria Baeza Pintado*, *Paloma Sanz Munoz*, *Ana Garcia Tello*, *Juan Boronat Catala*, *Juliusz Jan Szczesniewski*, *Jose Ramon March*, *Marina Garcia Cogollo*, *Cristina Hernandez Avila*, *Ainhoa Valle Rubio*, *Jose Luis Ramos Rodriguez*, *Alberto Hernandez Matias*, *Jose Mariá Jover Navalon* (**Hospital Universitario de Getafe**); *Mercedes Martinez Garcia*, *Celddy Mireya Urquizo Torrico*, *Silvia Benito Barbero*, *Justyna Drewniak Jakubowska* (**Hospital Universitario de Mostoles**); *David Stolle Duenas*, *Ana Nieto Moreno*, *Alfredo Abad Gurumeta*, *Paula Fernandez Valdes Bango*, *Alicia Ruiz Escobar*, *Diana Fernandez*, *Javier Ripolles Melchor*, *Elena Saez Ruiz*, *Elena Nieto Moreno*, *Norma Aracil Escoda*, *Elena Lucena De Pablo*, *Marina De Retes*, *Kateri Chao*, *Berenice Gutierrez Tonal*, *Eugenio Martinez Hurtado*, *Mercedes Garcia Dominguez*, *Barbara Algar Yanez*, *Paloma Munoz Saldana*, *Ana Maria Tirado Errazquin*, *Sandra Maria Gadin Lopez*, *Rut Salvachua*, *Beatriz Nozal*, *Cristina Sevilla Guillen*, *Rosa Sanz Gonzalez*, *Olga De La Varga Martinez*, *Marina Valenzuela Pena*, *Beatriz Vazquez Rivero* (**Hospital Universitario Infanta Leonor**); *Patricia Serrano-Mendez*, *Alexander Forero-Torres*, *Fuad Lopez-Fernandez*, *Francisco J Reinoso* (**Hospital Universitario La Paz**); *Clara Marchesi Alcober*, *Begona Ayas Montero*, *Sandra Verdeguer Ribes*, *Maria Jose Alberola Estelles (****Hospital Universitario y Politécnico La Fe****);* *Ana Navarro Barles*, *Xenia Carre Rubio*, *Ildefons Sarda Aure*, *Xenia Carre Rubio*, *Marta Silvestre Cepeda*, *Enrique Ruiz Velasquez (****Sant Joan de Reus University Hospital****);*

**Sudan:** *Sami Galal Eldin (****Al-Moalem Medical City****)*

**Syria:** *Wael Alkhaleel*, *Hala Alhomi Alsaid Mushaweh*, *Ahmad Alhaj*, *Marwan Al Aliwy*, *Marah Zeidani*, *Abdallah Aladna*, *Kusay Ayoub*, *Lama Kadoura*, *Mousa Sifat*, *Joudi Chachati*, *Ammar Niazi*, *Ehab Alaasy Alkushti*, *Hilal Matar*, *Abdalwahab Alkhalaf* (**Aleppo University Hospital**); *Opadah Salhab*, *Masa Watfa*, *Alhasan Alkhayer*, *Habib Olaisheh*, *Ali Hammed*, *Ahmad Molhem*, *Ali Sulaiman Deeb*, *Naya Hassan*, *Bassam Hassan*, *Ali Esmael*, *Hamza Ibrahim* (**Tishreen University Hospital**)

**Yemen:** *Zuhoor Almohanady*, *Ghadeer Al Wajeeh*, *Khadega Abotaleb*, *Waheeb Al Kubati*, *Ghofran Al Nahwi*, *Ashwaq Ziyad*, *Hanan Sheebren*, *Faris Alhajami*, *Hanadi Ali*, *Wejdan Al Wafi*, *Amani Altaweel*, *Belquis Aljailani*, *Salwa Al eryani*, *Abeer Mohammed*, *Abeer Saadan*, *Bushra Alshaikh*, *Shada Alemad*, *Manal Aldhaheri*, *Qasim Aldhaheri*, *Rafat Al Saban*, *Suhail Alshehab*, *Heba Abduljawad*, *Maha Muharram*, *Amal Muharram*, *Eman Al Markiz*, *Sarah Shream*, *Hanan Alhakimi*, *Shaima Ziyed*, *Ansam Alrobaiee Al Thawra (****Modern General Hospital****)*
